# Upregulation of a marine fungal biosynthetic gene cluster by an endobacterial symbiont

**DOI:** 10.1038/s42003-020-01239-y

**Published:** 2020-09-23

**Authors:** Mingwei Shao, Changli Sun, Xiaoxiao Liu, Xiaoxue Wang, Wenli Li, Xiaoyi Wei, Qinglian Li, Jianhua Ju

**Affiliations:** 1 CAS Key Laboratory of Tropical Marine Bio-resources and Ecology, Guangdong Key Laboratory of Marine Materia Medica, RNAM Center for Marine Microbiology, South China Sea Institute of Oceanology, Chinese Academy of Sciences, 164 West Xingang Road, Guangzhou, 510301 China; 2 College of Oceanology, University of Chinese Academy of Sciences, Beijing, 100049 China; 3 Southern Marine Science and Engineering Guangdong Laboratory (Guangzhou), Guangzhou, 511458 China; 4 Key Laboratory of Marine Drugs, Ministry of Education of China, School of Medicine and Pharmacy, Ocean University of China, Qingdao, 266003 China; 5 Laboratory for Marine Drugs and Bioproducts, Qingdao National Laboratory for Marine Science and Technology, Qingdao, 266237 China; 6 Key Laboratory of Plant Conservation and Sustainable Utilization, South China Botanical Garden, Chinese Academy of Sciences, Guangzhou, 510650 China

**Keywords:** Microbiology, Chemical biology

## Abstract

Fungal-bacterial associations are present in nature, playing important roles in ecological, evolutionary and medicinal processes. Here we report a fungus-bacterial symbiont from marine sediment. The bacterium lives inside the fungal mycelium yet is robust enough to survive independent of its host; the independently grown bacterium can infect the fungal host in vitro and continue to grow progenitively. The bacterial symbiont modulates the fungal host to biosynthesize a polyketide antimicrobial, spiromarmycin. Spiromarmycin appears to endow upon the symbiont pair a protective/defensive means of warding off competitor organisms, be they prokaryotic or eukaryotic microorganisms. Genomic analyses revealed the spiromarmycin biosynthetic machinery to be encoded, not by the bacterium, but rather the fungal host. This unique fungal-bacterial symbiotic relationship and the molecule/s resulting from it dramatically expand our knowledge of marine microbial diversity and shed important insights into endosymbionts and fungal-bacterial relationships.

## Abstract (web summary)

Shao et al. show that a bacterial symbiont drives its fungal host to biosynthesize a polyketide antimicrobial, spiromarmycin, fending off their competitors. They find that the spiromarmycin biosynthetic machinery is encoded by the fungal host. This study provides insights into the evolution of marine microbial diversity.

## Introduction

Bacteria occupy diverse ecological niches and build strong mutualistic associations with a myriad of other organisms^1^. Endosymbiotic bacteria residing in animals^2–4^, plants^5^, insects^6^, and worms^7^ are well known. However, reports of symbiotic endofungal bacteria are less ubiquitous^8–11^; the *Burkholderia rhizoxinica* (Mucoromycotina)^8–10^ and Arbuscular mycorrhizal endofungal bacteria^11^, are a few of these rare examples. Despite limited studies, advances in our understanding of fungus-associated microbes have been made, especially in the context of plant epiphytic fungi. Initiatives to identify and exploit novelty in fungal/endobacterial couplings have started to focus on the impact that different habitats have upon such symbiotic relationships^12^. Central to this idea, the marine environment represents an excellent reservoir of symbiotic associations able to not only withstand, but to actually benefit, from the intense ecological and evolutionary pressures presented by life in the oceans^13,14^. Yet, to date, there have been no reports of marine-derived fungal-endobacterial pairings. We report here, the discovery and identification of a fungal-endobacterial symbiont SCSIO F190/B001 from a marine sediment sample. The endobacteria modulates the fungal host to biosynthesize a polyketide natural product, spiromarmycin, whose structural elucidation, biosynthetic machinery and diverse biological activities, we also present.

## Results and discussion

### Isolation of fungal-endobaterial symbiont SCSIO F190/B001

**Fig. 1:**
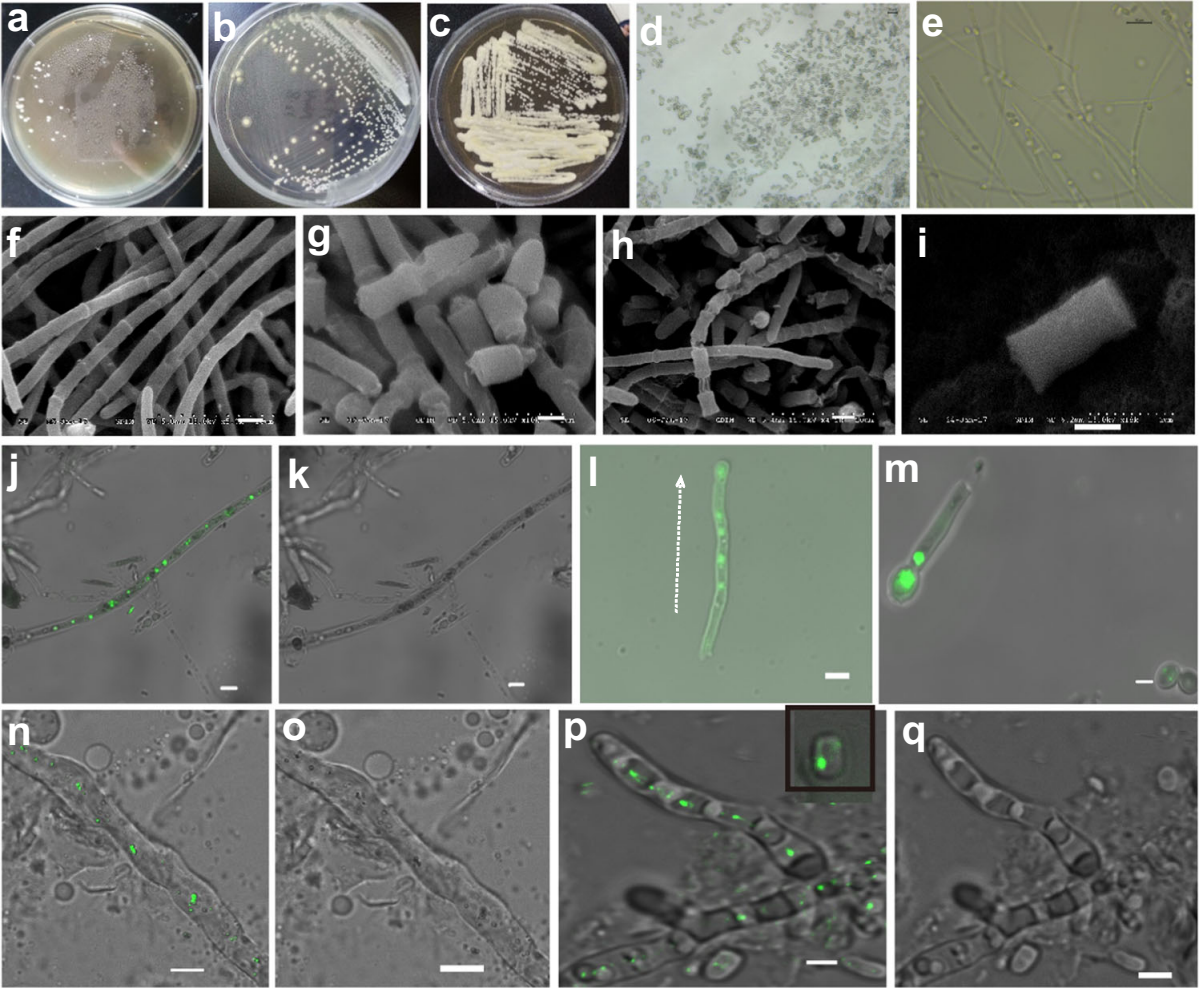
Morphological identification of symbiont F190/B001. **a**–**c** The morphology of the symbiont F190/B001 on the ISP2 plate. **d**–**e** The morphology of arthrospore and mycelium under optical microscope (400*). **f**–**h** The morphology of the symbiont F190/B001 mycelium after growth on the ISP2 plate (without antibiotics) for 7 days shows a filamentous septate hyaline to brown hyphae with a section able to form the spore. **i** The morphology of spore, which shows the spore is septate, cylindrical, and straight. **j**–**m** Mycelium and spore of symbiont F190/B001 were stained with SYTO 9 and observed using a Leica TCS SP8 AOBS Laser Microscope at 510/520 nm (fluorescence mode). Green fluorescence in the fungal hyphae or arthrospore indicates that the mycelia harbored a large number of alive endobacteria. **n**–**o** Fluorescent in situ hybridization (FISH) micrographs of symbiont F190/B001 mycelium observed using a Leica TCS SP8 AOBS Laser Microscope at 515/530 nm (fluorescence mode). **p**–**q** Confocal Laser Scanning micrographs of symbiont F190/B001 mycelium after coculture with GFP-Labeled endobacterium *A. faecalis* SCSIO B001. The fungal hyphae or spore contain a rod-shaped GFP-labeled endobacterium (fluorescence mode). Scale bar, 1 µm.

To recapitulate the actinomycete richness of the oceanic environment, a sediment sample collected from the northern South China Sea was diluted and coated onto a fucose-proline agar plate containing the antibacterial trimethoprim and antifungal nystatin. After 2 weeks, a single colony was isolated and the purity of the culture was ensured by three rounds of purification using different media; ISP2, malt extract agar medium and potato dextrose agar containing trimethoprim were successively employed to ensure the purity of the isolated microorganism. Interestingly, the purified colony indeed assumed the visual characteristics of an actinomycete in its early stages of growth (Fig. 1a). However, over time, the colony assumed more of a fungal appearance (Fig. 1c). To classify the taxonomy of the isolated microorganism, the genetic markers, bacterial 16S rRNA gene and fungal ITS1-5.8S rDNA-ITS2 region were amplified by PCR and subsequently sequenced by Sanger sequencing to test the genomic DNA of the isolated microorganism. Notably, the 16S rRNA gene test was positive and a single strip of ITS1-5.8S rDNA-ITS2 amplicons was also obtained (Supplementary Fig. 1). The 16S rRNA gene sequence showed a high degree of similarity (99%) to bacterium *Alcaligenes faecalis* whereas the ITS1-5.8S rDNA-ITS2 sequence showed 96% sequence similarity and 93% query coverage to fungal *Spiromastix* sp. CBS13827 sequences identifiable by BLASTN. Hence, the culture possesses both bacterial and fungal signatures. On the basis of these findings, we named the microbe pairing fungus-bacterium symbiont SCSIO F190/B001.

Early morphological investigations of SCSIO F190/B001 revealed that colonies grown for a period of 4–7 days on ISP2 medium devoid of antibiotics assumed the appearance of a white powdery surface. The symbiont could be easily cultured and, like actinomycetes, did not disperse (Fig. 1a, b). Over time, a nondiffuse light brown pigment became evident (Fig. 1c) and at 7 days was subjected to scanning electron microscopy. A filamentous septate hyaline to brown hyphae with a section able to form the spore (Fig. 1f–h) was clearly apparent via scanning electron microscopy. The spore was septate, cylindrical, and straight, measuring 2.9–3.0 × 1.4–1.5 µm (Fig. 1i). These morphological characteristics were consistent with identification of the fungus as a *Spiromastix* sp.^15^. Surprisingly, resistance screens revealed the fungus-bacterium pairing to be resistant to all antimicrobial agents employed during purification; kanamycin, nystatin, geneticin, and hygromycin all proved ineffective at inhibiting symbiont growth (Supplementary Table 1).

### Mutualistic associations of symbiont SCSIO F190/B001

The duality of both fungal and bacterial characteristics displayed by the symbiont suggested the presence of an endosymbiotic bacterium housed within the fungal host. Accordingly, we attempted to acquire the pure bacterial component by fungal mycelium lysis/disruption and subsequent spreading of the mycelium contents onto Luria-Bertani (LB) medium plates (see methods). Ultimately, only one bacterial species was acquired and then subjected to morphological and molecular biological analyses. In line with earlier 16S rRNA gene sequencing data for the intact symbiont pairing, the bacterium was identified as *A. faecali* SCSIO B001 (Supplementary Figs. 2 and 4). In contrast to earlier resistance screens for the symbiont SCSIO F190/B001, the purified *A. faecali* SCSIO B001, in the absence of its fungal host, was highly sensitive to the full panel of antibacterial agents assayed (see Supplementary Table 1), suggesting that an important aspect of the proposed F190/B001 mutualism may involve fungal protection of its bacterial inhabitant.

Importantly, symbiont staining studies with SYTO-9 and propidium iodide^8^ that is specific for bacteria as well as FISH studies (see methods) using the BET42a probe^16^, a universal 23S rDNA probe labeled with a 5′-6-carboxy tetramethylrhodamine fluorescein tag targeted to the *A. faecali* SCSIO B001 genome revealed that *A. faecali* SCSIO B001 resides predominantly within the mycelia of the fungal host (Fig. 1j–o). Furthermore, we have also shown that the fungal hyphae cells contain the rod-shaped GFP-labled endobacterium B001 by cocultivation of the symbiont F190/B001 with GFP-labeled *A. faecalis* SCSIO B001 (Fig. 1p, q). These observations provided strong evidence that transmission of the bacterial component of the F190/B001 symbiont may occur horizontally by means of infection in vitro. In addition, coculture of GFP-labled endobacterium B001 with the symbiont F190/B001 also revealed that *A. faecali* SCSIO B001 could reside within the fungal spores (Fig. 1p), indicating that the bacterial component of the F190/B001 symbiont may be transmitted vertically through spores of its fungal host. *A. faecali* SCSIO B001 not only could enter the fungal spores thus transferring to the next generation, but also can be released from the fungal host cell and reinfect the fungal host, seemingly a parasitism-to-mutualism shift^17,18^.

In rounding out our early characterization of the F190/B001 mutualistic association, we next sought to obtain endobacterium-free *Spiromastix* sp. SCSIO F190. We employed several approaches to remove the *A. faecali* SCSIO B001 component from the fungal host including subjection to assorted antibiotics as well as protoplast regeneration strategies (see “methods”). However, this challenge proved insurmountable; viable *Spiromastix* sp. SCSIO F190 was found to display a pronounced endobacterial dependence (Supplementary Fig. 5m, n). This finding, in particular, speaks strongly in favor of there being a mutualistic symbiosis between the fungal host and its endobacterium.

### Symbiont SCSIO F190/B001 produces spiromarmycin

**Fig. 2:**
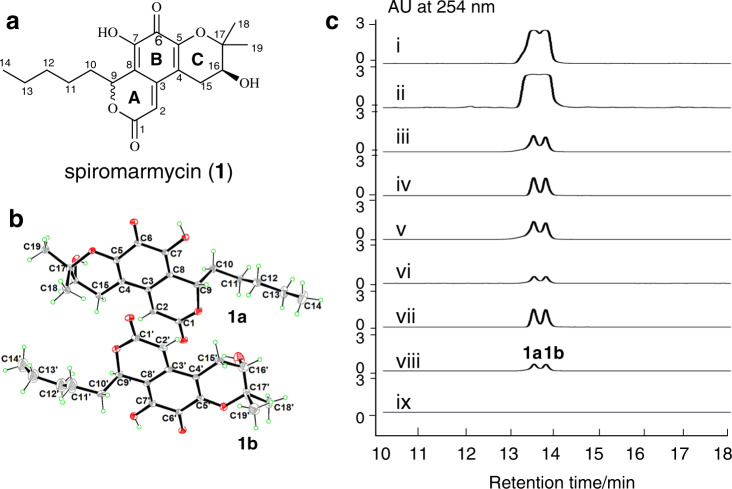
Fungal production of spiromarmycin (1) is triggered by endobacterium. **a** The structure of spiromarmycin (**1**). **b** X-ray structure of spiromarmycin (**1**). **c** HPLC analyses of the PDA fermentation butanone extracts. (i: negative controls of the symbiont F190/B001; ii: symbiont F190/B001 treated with ampicillin; iii symbiont F190/B001 treated with kanamycin; iv: symbiont F190/B001 treated with chloromycetin; v: symbiont F190/B001 treated with ciprofloxacin; v: symbiont F190/B001 after treated with four kinds of antibiotics and retrained on a ISP2 plate containing ciprofloxacin hydrochloride consecutively; vii: symbiont F190/B001 precipitate out the irregular brown plaques; viii: purified compounds spiromarmycin; ix: *A. faecalis* SCSIO B001; mAU, milliabsorbance units).

Butanone extractions of F190/B001 fermentations afforded a crude extract with good antibacterial activities against several clinical pathogenic bacteria [Methicillin-resistant *Staphylococcus aureus* (MRSA), *Enterococcus faecium*, *Micrococcus luteus*, *Bacillus thuringiensis*] without detectable resistance compared with ampicillin, kanamycin and blank control (Supplementary Fig. 5c). Ultimately, bioassay-guided fractionations allowed us to correlate antibacterial activities to a compound with molecular formula C_19_H_24_O_6_ (Supplementary Fig. 6 and 7). The structure of this antimicrobial, herein named spiromarmycin (**1**), was established by X-ray crystallography of a crystal grown from acetone-methanol-water (Fig. 2a, b).

Spiromarmycin (**1**) was found to crystallize as two molecules in one asymmetric unit with the same planar structure but different absolute configurations at C-9. HPLC analyses of crystalline **1** revealed the presence of two peaks (an elution system consisting of CH_3_CN/H_2_O), **1a** and **1b**, indicating that **1** exists as an equilibrating mixture of two different isomers. Careful semi-prep HPLC enabled the separation of **1a** and **1b**. Although **1a** and **1b** were found to be stable in CHCl_3_ over the course of 12 h, the use of protic solvents such as MeOH and H_2_O enabled moderately fast equilibration, *via* a net C-9 epimerization, over the course of 8 h (see Supplementary Tables 2–3 & Fig. 8). The 1D and 2D NMR data in CDCl_3_ of **1a** and **1b**, together with their rotation, and circular dichroism (CD) spectra, were recorded immediately following seperation. A full summary of NMR shifts and assignments are provided in Supplementary Table 4. The results of electronic circular dichroism (ECD) analyses for **1a** and **1b** were in good agreement with the calculated ECD data for 9*S*, 16*S* and 9*R*, 16S structures, respectively (see Supplementary Fig. 9).

We envision that the unique structure of **1** is amenable to moderately rapid epimerization at C-9 by virtue of C-9’s unique vinylic and highly conjugated nature. Both the lactone (ring A, Fig. 2a) and α-hydroxy enone (ring B, Fig. 2a) are in conjugation with each other, and able to transiently assume various tautomers that effectively acidify the C-9 proton; this enables transient planarity at C-9 via deprotonation. In particular, access to C-9 vinylic intermediates **1c** (Supplementary Fig. 8), especially in protic solvents, likely explains the presence of the noted 1:1 ratio of **1a** and **1b** encountered during HPLC analyses of **1**.We envision that **1**, in protic solvents, is simply prone to C-9 epimerization as a result of its unique positioning relative to rings A and B of spiromarmycin. In truth, ^1^H NMR spectroscopic data of D_2_O-incubated **1a** revealed the retention of the C-9 hydrogen (Supplementary Fig. 10), supporting the mechanism mentioned above for C-9 epimerization and interconversion of **1a** and **1b**.

### Antimicrobial activity of spiromarmycin

Marine microorganisms from sediment must not only endure harsh conditions, but also face intense competition for often exceedingly scarce nutrients. As such, it is no surprise that such organisms have evolved diverse antimicrobial mechanisms to ensure survival and protection from competitors. This logic inspired us to assay spiromarmycin’s activities against a panel of 58 drug resistant bacteria (29 Gram-negative and 29 Gram-positive), 8 *Candida albicans* strains isolated from different sources and 21 plant pathogenic fungi. In addition, the activity of spiromarmycin against the isolated endobaterium *A. faecali* SCSIO B001 was also tested.

This campaign revealed **1** as a diverse antimicrobial; spiromarmycin possessed not only good antibacterial activities but also good antifungal activities (see Tables 1 and 2; a complete summary of all bioactivity data generated for **1** is shown as Supplementary Tables 5 and 6). Notablely, spiromarmycin was found to have no antibacterial activity against *A. faecali* SCSIO B001.

**Table 1:** Selected MIC values of spiromarmycin (**1**) against prokaryotic and *Candida albicans*(in μg/mL).

Organism	Source	MIC (μg/mL)
*Staphylococcus aureus* ATCC 29213	Clinically resistant bacteria	4.0
MRSA /methicillin-resistant *Staphylococcus aureus*	Clinically resistant bacteria	16.0
*Staphylococcus aureus* (cfr) GDQ6P012P	Poultry pathogen	16.0
*Vibrio alginolyticus* XSBZ14	Algicidal bacteria	32.0
*Enterococcus faecalis* ATCC 29212	Clinically resistant bacteria	16.0
*Enterococcus faecium* 36235	Clinically resistant bacteria	16.0
*Micrococcus luteus*	Clinically resistant bacteria	8.0
*Bacillus thuringiensis*	Clinically resistant bacteria	16.0
*Acinetobacter baumannii* ATCC 19606	Clinically resistant bacteria	128.0
*Escherichia coli* 16369	Clinically resistant bacteria	128.0
*Candida albicans* ATCC96901	Clinically resistant strain	16.0
*Candida albicans* 173202375	Clinically resistant strain	2.0
*Candida albicans* 174105146	Clinically resistant strain	1.0

**Table 2:** IC_50_ Values for spiromarmycin activity against eukaryotic microorganisms (in μg/mL)^a^.

Phytopathogen	Spiromarmycin	Cycloheximide^b^	Metalaxyl^b^	Dimethomorph^b^
*Rhizoctonia solani*	3.2 ± 0.6	0.3 ± 0.0	NT	NT
*Valsa mali*	4.3 ± 1.1	0.3 ± 0.0	NT	NT
*Gibberella sanbinetti*	5.7 ± 0.3	3.3 ± 0.2	NT	NT
*Fusarium* spp	8.1 ± 0.5	2.6 ± 0.1	NT	NT
*Dothiorella gregaria*	>50 ± 0	0.9 ± 0.1	NT	NT
*Altemaria solani*	1.2 ± 0.1	4.4 ± 0.2	NT	NT
*Fusarium. oxysporum* f. sp. *Cucumerinu*	5.0 ± 0.4	2.3 ± 0.3	NT	NT
*Fusarium oxysporum* f. sp. *momordicae*	>50 ± 0	10.6 ± 1.0	NT	NT
*Fusarium oxysporum* f. sp. *Vasinfectum*	>50 ± 0	1.7 ± 0.1	NT	NT
*Gibberella zeae*	>50 ± 0	4.9 ± 0.2	NT	NT
*Helminthosporium maydis*	>50 ± 0	>50 ± 0	NT	NT
*Botrytis cinerea* Pers	17.1 ± 0.8	28.8 ± 8.7	NT	NT
*Physalospora piricpla Nose*	>50 ± 0	4.3 ± 0.7	NT	NT
*Colletot tichum gloeosporioides Penz*	3.5 ± 0.6	1.9 ± 0.9	NT	NT
*Colletot tichum gloeosporioides*	1.8 ± 0.6	0.8 ± 0.3	NT	NT
*Ceratobasidium cornigerum*	1.7 ± 1.1	0.02 ± 0.0	NT	NT
*Bipolaris sorokiniana*	>50 ± 0	8.5 ± 0.9	NT	NT
*Penicillium digitatum*	>50 ± 0	>50 ± 0	NT	NT
*Phytophthora capsici* LT1534	8.1 ± 0.7	NT	38.7 ± 5.9	0.09 ± 0.0.02
*Phytophthora capsici* P35	4.5 ± 0.3	NT	1.4 ± 0.1	0.6 ± 0.2
*Phytophthora capsici* LT263	1.5 ± 0.3	NT	11.1 ± 1.5	0.5 ± 0.04

*NT* No tested.

^a^Values represent the mean of three replications ± standard deviation.

^b^Cycloheximide was coassayed as a positive control.

### Genome analyses of symbiont SCSIO F190/B001

To understand the biosynthesis of spiromarmycin as well as the genetics and relationship of *A. faecali* SCSIO B001 and symbiont F190/B001, the complete genome of *A. faecali* SCSIO B001 was sequenced. The complete genomic features of endobacterium *A. faecali* SCSIO B001 are summarized in Supplementary Table 7 and display a high degree of similarity to *Alcaligenes* sp. ZD02^19^ in terms of G + C content (56.82%), gene numbers (3752), tRNA (57) and, rRNA (9). However, *Alcaligenes* sp. ZD02 contains a 14,928 bp plasmid, and a larger genome size (4233756 bp); the genome size of *A. faecali* SCSIO B001 is 4029778 bp. Interestingly, no plasmid from endobacterium *A. faecali* SCSIO B001 was detected (Supplementary Fig. 11).

Notably, it has been reported that fungal endobacteria undergo reductions in genome size relative to their “non-endobacterial counterparts”; this is especially pronounced in endosymbionts that are uncultivatable as standalone organisms and that display host dependencies^20^. For example, endobacteria of *Glomeromycotina* (arbuscular mycorrhizal fungi) have reduced genome sizes [*Candidatus* Glomeribacter gigasporarum genome (1.7-1.9 Mb)^21^ and *Candidatus* Moeniiplasma glomeromycotorum genome (0.7–1.3 Mb)^22,23^] and strict host dependencies. Alternatively, the endosymbiont *B. rhizoxinica* (3.7 Mb)^24^ and *Mycoavidus. cysteinexigens* (~2.6 Mb)^25,26^, with their larger genomes, can grow quite well independent of any fungal host. Interesting from the perspective of the current work is that *Alcaligenes* sp. SCSIO B001 has been isolated from the symbiont F190/B001 successfully and appears to lack any obvious host dependencies. However, our efforts to obtain the endobacterium-free *Spiromastix* host have failed suggesting that the host is more dependent upon its inhabitant than is ordinarily the case of fungal-bacterium symbiont systems.

Since no symbiont-free fungal *Spiromastix* sp. SCSIO F190 could be obtained, we cultured symbiont F190/B001 in the presence of different antimicrobial agents (including ampicillin, kanamycin, chloromycetin, and ciprofloxacin hydrochloride). Total genomic DNA from antimicrobial-treated F190/B001 was extracted and sequenced. The genome of *Spiromastix* sp. SCSIO F190, following subtraction of B001 genome data, was found to comprise 38.2 M bp of information with the G + C content of 44.76% (Supplementary Table 8). The GC content for antimicrobial-treated F190/B001 proved much more readily decipherable than that of the genome for antimicrobial-untreated F190/B001 (Supplementary Fig. 12), suggesting a bacterial role in F190 genomic maintenance. These results also suggested that although the endobacterium B001, when existting as part of the symbiont, is conferred resistance to antimicrobials as revealed by earlier resistance screens of the symbiont F190/B001, this resistance is not vigorous enough to protect the endobaterium B001 to avoid the suppression in some extent by the antimicobials. Indeed, this observation is consistent with our earlier efforts to generate SCSIO F190 completely devoid of its endobacterial partner. We then performed comparative analyses of the acquired F190 genome against those of ten fungi strains with relatively close relatives to the symbiont F190/B001 isolate in the ITS1-5.8S rDNA-ITS2 sequence-based phylogenetic trees. Genomic sequence-based analysis revealed that SCSIO F190 is located in different branches, between *Coccidioides immitis* RS and *Emmonsia crescens* UAMH4076 in the genomic evolution tree (Supplementary Fig. 13) and strongly suggested that SCSIO F190 represents a putatively new species of termed *Spiromastix* sp. Further protein sequence comparisons for *Spiromastix* sp. SCSIO F190 and these selected ten fugal strains by cd-hitsoftware, enabled us to generate a Core-Pan gene summary as shown in Supplementary Figs. 14 and 15. Dilution curves were obtained by analyzing different combinations of core and pan genes; progression through these dilution curve revealed that core genes become less frequent with increasing species whereas the pan gene set increases with increasing number of species detailed (Supplementary Figs. 14). These results revealed that the *Spiromastix* sp. SCSIO F190 genome encodes for 3157 specific genes and only 761 core genes (Supplementary Fig. 15), suggesting that *Spiromastix* sp. SCSIO F190 is markedly different from other fungi at the genomic level. These data further validate the notion that SCSIO F190 is a putatively new fungal species. To our knowledge this is the first report of a fungal genome sequence of the *Spiromastix* genus.

### Fungal spiromarmycin construction

To further understand spiromarmycin biosynthesis, genomic data for both *A. faecali* SCSIO B001 and *Spiromastix* sp. SCSIO F190 were analyzed using online antiSMASH software. Bioinformatics analyses of *A. faecali* SCSIO B001 revealed only one type I PKS gene cluster (Supplementary Table 10) in the bacterial genome. The limited functionality encoded by this PKS cluster (Supplementary Table 10) suggests its inability to generate the complete C_19_ backbone of spiromarmycin. At this stage it was evident that the bacterially encoded PKS in the F190/B001 symbiont pairing is unable to produce spiromarmycin. This finding was further supported by HPLC-based metabolomics assays with *A. faecali* SCSIO B001, which revealed the complete absence of spiromarmycin in bacterial fermentations (Fig. 2c, trace ix). In tandem, bioinformatics analyses of both organisms suggested that spiromarmycin might be produced by the *Spiromastix* sp. SCSIO F190.

That fungal production of spiromarmycin might be triggered by *A. faecali* SCSIO B001 was suggested during early morphological studies of the F190/B001 symbiont treated with and without the antibacterial agent ciprofloxacin. Symbiont F190/B001 not subjected to ciprofloxacin was characterized by irregular brown plaques that were absent in symbiont samples treated with antimicrobial agents (Supplementary Fig. 5a, b); subsequent analyses revealed these plaques to correspond to spiromarmycin isomers **1a** and **1b** (Fig. 2c, trace vii). We hypothesized that the killing of *A. faecali* SCSIO B001 (by antimicrobials like kanamycin, ciprofloxacin and others) stimulated decreased production of spiromarmycin, presumably by the still viable fungal host. This was validated, in part, by assays in which the F190/B001 symbiont was grown either in the presence or absence of antimirobials (ampicillin, kanamycin, chloromycetin, and ciprofloxacin hydrochloride). These side-by-side fermentations and subsequent metabolite analyses revealed that deleterious effects upon F190 by these antimicrobial agents actually led to moderately decreased yields of spiromarmycin (Fig. 2c, trace i–v). A reasonable explanation for these decreased yields of sipromarnycin is that the antimicrobial agents in the medium suppress most of bacterium component (both the bacteria attached to the surface of the fungal hyphae and the endobacteria in the fungal cell) but could not completely kill the bacterium component (as revealed by morphological studies and genome analyse of symbiont F190/B001, Supplementary Fig. 12), leading to the decreased quantity of alive endobacterium component of symbiont F190/B001; the resulting decreased trigger effect by endobacterium lead to the decreased spiromarmycin production by the still viable fungal host. These data, combined with scarcity of BGCs within the *A. faecali* genome, support our hypothesis that the B001 is not the producer of spiromarmycin, but rather, a trigger for its production by the fungal host.

**Fig. 3:**
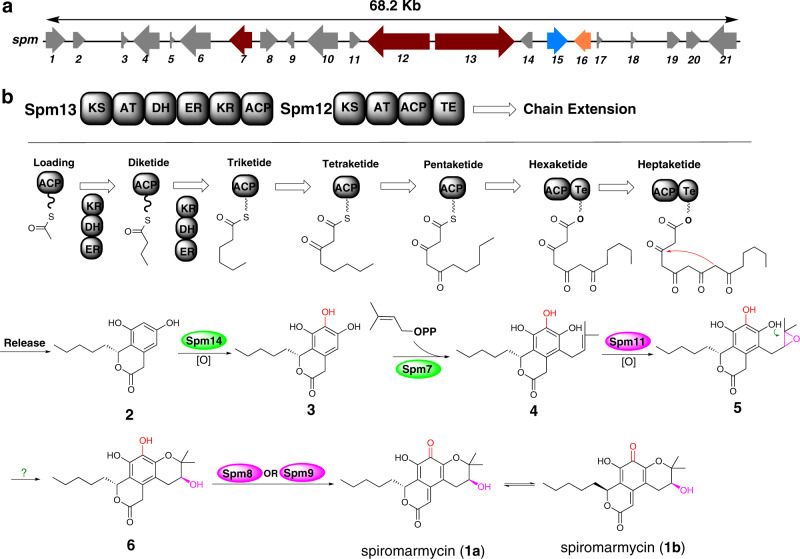
Postulated biosynthesis of spiromarmycin (1). **a** Genetic organization of the spiromarmycin biosynthetic gene cluster. **b** According to the ^13^C-labeling experimental results and bioinformatics analysis, we propose a reasonable biosynthetic pathway of spiromarmycin.

Bioinformatics analyses of the complete genome of *Spiromastix* sp. SCSIO F190 revealed the presence of 11 putative PKS gene clusters. Among these, one PKS gene cluster (Supplementary Table 11), containing 21 open reading frames and spanning 68.2 Kb of contiguous genomic DNA, was proposed to constitute the putative spiromarmycin gene cluster (termed herein *spm*, Fig. 3a). Assignment of the *spm* cluster was based partly on the clarity with which deduced gene products could be assigned functions using homologous systems (Supplementary Table 11). The *spm* cluster contains one fungal reducing iterative PKS gene, *spm13*, and one fungal nonreducing iterative PKS gene, *spm12*. The reducing iterative PKS, Spm13, consists of ketosynthase (KS), acyltransferase (AT), dehydratase (DH), enoyl reductase (ER), ketoreductase (KR), and acyl carrier proteins (ACP) domains whereas the nonreducing iterative PKS, Spm12, was found to contain only KS, AT, ACP, and thioesterase (TE) domains (Fig. 3b). Feeding experimemts using ^13^C-lablled building blocks [1-^13^C]acetate and [2-^13^C]acetate led to signal increase at all 19 carbon actoms of spiromarmycin (**1**) (Supplementary Figs. 16–20), demonstrating that the spiromarmycin (**1**) are indeed PKS-derived species.

Careful consideration of the intact *spm* cluster suggests that first, the reducing iterative PKS Spm13 may catalyze chain priming and operate iteratively to condense two malonyl-CoA equivalents to yield the triketide; after each condensation catalyzed by the KS domain, the polypeptide tethered to the ACP domain is subjected to β-ketoreduction by the KR domain, β-dehydration by the DH domain and α-β-enoylreduction by the ER domain. After formation of the triketide, further chain extension is proposed to be catalyzed by the nonreducing PKS Spm12 operating iteratively to catalyze four round of condensation to yield the heptaketide. Following liberation from the PKS by the TE domain of PKS Spm12, we envision that the putative hydroxylase (encoded by *spm14*) catalyzes C-6 hydroxylation of the pentyl-fused benzopyrone substrate **2**. Triol intermediate **3** then likely serves as a substrate for C-4 prenylation by the putative prenyltransferase Spm7; epoxidation of the terminal olefin by Spm11 is readily envisioned to give way to intramolecular etherification (generating ring C of **1**) and concomitant epoxide ring opening. The resulting intermediate, catechol **6** (Fig. 3b) is then readily oxidized, most likely by is oxidized by the FAD-dependent monooxygenase encoded by *spm8* to natural product **1**. Notably, as discussed above, the unique fusion and functionalities of rings A and B of **1** make this compound highly amenable to non-enzymatic epimerization at C-9. Thus, in the presence of protic solvents, **1** is found to exist as a generally 1:1 mixture of diastereomers **1a** and **1b**.

Having identified the fungal spiromarmycin gene cluster, we next sought to further confirm the activation of the identified *spm* gene cluster by the presence of the endobaterium B001. The quantitative real time RT-PCR analysis revealed that the relative level of the transcripts of two PKS genes, *spm12* and *spm13*, in the antimicrobial-treated F190/B001 was substantially lower than that in the antimicrobial-untreated F190/B001 (Supplementary Fig. 21). These results indicated that the negative effects of the antimicrobial compounds on the endobaterium led to the downregulation of the identified gene cluster and further supported our hypothesis that the endobacterium is indeed a trigger for its spiromarmycin production by the fugal host.

Earth’s oceans have likely served as the cradle of life, yet, until now, there have been no reports of marine-based fungal-endobacterium associations. We report here, a marine fungal-bacterium symbiont isolated from the South China Sea, to our knowledge, the first of its kind. This symbiotic pairing was identified on the basis of comparative morphology and molecular biological studies. We found that *Spiromastix* sp. SCSIO F190 is a putatively new strain of the genus *Spiromastix*, which currently has only six species^27^, including *S. warcupii*, *S. grisea*, *S. tentaculata*, *S. saturnispora*, *S. princeps* and *S. sphaerospora*. This advance was enabled by our sequencing of the SCSIO F190 genome, the first *Spiromastix* genome sequenced. The bacterial symbiont was identified as endobacterium *A. faecali* SCSIO B001; GFP labeling indicates that SCSIO B001 mainly colonizes the interior of the fungal host.

**Fig. 4:**
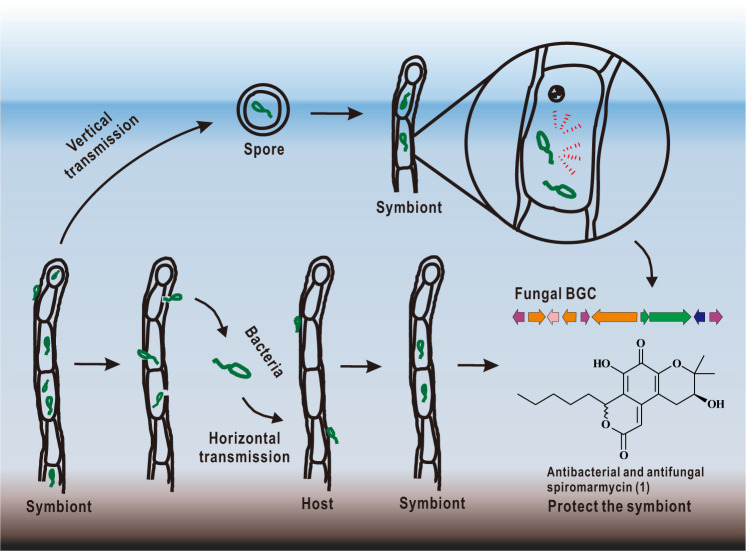
Model for mutualistic associations of symbiont SCSIO F190/B001. The endobecteria *A. faecali* SCSIO B001 can be released from the fugal host cell and infect the fungal host cells in vitro and continue to grow progenitive; endobecteria *A. faecali* SCSIO B001 can also enter the vegetative fungal spores and are transferred to the next generation. The endobecteria modulates fungal host to produce the broad spectrum antimicrobial spiromarmycin, which can help the symbiont drive away the surrounding microbes (prokaryotic or eukaryotic microorganisms) to gain more living conditions in marine enviroment.

Data presented here support the notion that the *Spiromastix* host and *A. faecali* SCSIO B001 behave as mutualistic symbionts (Fig. 4). By serving as the bacterial host, the fungus appears to gain a selective advantage over competing organisms by producing the broad spectrum antimicrobial spiromarmycin; early studies suggest that the *spm* cluster, housed within the fungal genome, is modulted by the B001 bacterium. Also clear from these studies is that the *Spiromastix* host is readily infected, or reinfected based on specific scenarios, by the B001 bacterium and also is able to provide an ideal intracellular habit. In sum, what is clear from these experiments is that both organisms gain a selective advantage by teaming up and that both horizontal and vertical transmissions play a central role in the F190/B001 symbiosis.

## Methods

### Isolation of symbiont SCSIO F190/B001

The symbiont F190/B001 was isolated from a marine sediment sample collected in the Northern South China Sea. Briefly, the sample was homogenized, and serial dilutions (10^−1^, 10^−2^, 10^−3^) were streaked onto fucose-proline agar (FPA) medium (consisting of 1 g proline; 5 g fucose; 1 g K_2_HPO_4_; 1 g MgSO_4_.7H_2_O; 1 g (NH_4_)_2_SO_4_; 1 g multi-vitamin; 30 g baysalt; 2 g CaCO_3_; 20 g agar in 1 L of distilled water; pH 7.0-7.4) plates containing trimethoprim and nystatin at concentrations of 50 μg/mL. The plates were incubated aerobically in a chamber for 14 d at 28 ± 0.5 °C and colonies were transferred and purified on another FPA medium plates to obtain pure cultures. After 3 generations of purifications, the isolated microbe was preserved on FPA medium slants at 4 °C until used.

### Morphological observations of symboint F190/B001

To observe the morphology of the microorganism, the symboint F190/B001 was purified by different culture medium including ISP2 (4 g glucose; 4 g yeast extract; 10 g malt meal; 20 g agar in 1 L of distilled water; pH 7.2 − 7.4), malt extracts agar medium and potato dextrose agar containing trimethoprim. The purified single clone grown on ISP2 medium plates without antibiotics was prepared for the observation of light microscope and the scanning electron microscope.

### Isolation of endobacterium SCSIO B001

To get the endosymbiotic bacterium, the symbiont F190/B001 was first grown on potato dextrose agar medium plates for 3 weeks and then the mycelium was collected and sheared by pipetting. The broken mycelia were spread on the LB medium plates and incubated aerobically in a chamber at 28 ± 0.5 °C.

### Regeneration of protoplast of symbiont F190/B001

In an attempt to get symbiont-free fungal strain *Spiromastix* sp. SCSIO F190, we performed the preparation and regeneration of protoplast of symbiont F190/B001^28^. The spore of symbiont F190/B001 was inoculated into the 10 mL potato dextrose broth medium and cultivated for 2 days at 28 °C and 150 rpm. The mycelia were transferred into 100 mL potato dextrose broth medium and allowed to grow for 3 days at 28 °C and 150 rpm and then collected by filtration. The mycelia were digested using 1% Yatalase (Takara) in 0.6 M (NH_4_)_2_SO_4_, 50 mM maleic acid, pH 5.5 at 30 °C for 5 h. The residues were then removed by filtration, and the protoplasts were centrifuged at 1500 rpm for 10 min and washed with solution 2 (1.2 M sorbitol, 50 mM CaCl_2_·2H_2_O, 35 mM NaCl, 10 mM Tris-HCl, pH 7.5). The protoplasts were collected and adjusted to 1 × 10^6^ cells mL^−1^ in Solution 2. Mixtures of 200 μL protoplasts solution were spread on the lower potato dextrose agar medium (containing 0.7 M NaCl as well as 50 μg/mL ciprofloxacin hydrochloride, pH 5.5) with 1.5% agar, and then covered with the same potato dextrose agar medium as described above (containing 0.8% agar). The plates were incubated at 30 °C for 2–5 days.

### DNA extraction, PCR, and sequencing

In preparation for DNA extraction, symbiont F190/B001 was grown on ISP2 agar plates containing trimethoprim and nystatin at 50 μg/mL. The fresh mycelia grown on ISP2 agar medium at 28 °C for 7 days were inoculated into 250 mL Erlenmeyer flask containing 100 mL of trypticase soy broth medium. After 3–4 days of incubation at 28 °C on rotary shakers at 200 rpm, thallus was used as samples for total DNA extraction. The genomic DNA was extracted with the E.Z.N.A. SP Fungal DNA Mini kit (Omega Bio-tek, Inc) according to the manufacturer’s instructions. The extracted DNA was stored at −20 °C until further use.

The ITS1-5.8S rDNA -ITS2 region were amplified using the primers of ITS1 (5′-TCCGTAGGTGAACCTGCGG-3′) and ITS4 (5′-TCCTCCGCTTATTGATATG-C-3′)^29^. Amplification was performed in a 50 μl reaction mixture containing: 1 µL of genomic DNA (~50 ng), 0.5 µL of FastPfu DNA Polymerase (1 U) (Transgen Biotechnology Co., Ltd., Beijing, China), 5 µL of reaction buffer (10×) and 5 µL of dNTPs (2.5 mM each) and 1 µL of each primer (10 µM) and 36.5 μL of sterile Milli-Q water. The PCR conditions: an initial denaturation step at 95 °C for 5 min, followed by 35 cycles of 60 s denaturation at 95 °C, 60 s primers annealing at 55 °C, and 30 s extension at 72 °C, and 10 min at 72 °C for a final chain elongation. The size and purity of PCR products were evaluated by 1% agarose gel electrophoresis.

Samples of 16S rRNA gene were amplified using the primers 27 F (5′-AGAGTTTGATCCTGGCTCAG-3′) and 1492 R (5′-GGTTACCTTGTTACGACTT-3′) based on the total genomic DNA. Amplification was accomplished in a 50 µL reaction mixture containing 1 µL of genomic DNA (~50 ng), 0.5 µL of FastPfu DNA Polymerase (1 U) (Transgen Biotechnology Co., Ltd., Beijing, China), 5 µL of reaction buffer (10×) and 5 µL of dNTPs and 1 µL of each primer, and 36.5 µL of sterile Milli-Q water. The PCR conditions were 30 cycles at 95 °C for 2 min, 55 °C for 1 min, and 72 °C for 2 min and 10 min at 72 °C for a final chain elongation. PCR products of the expected size (~1.5 kb) were evaluated on a 1% agarose gel electrophoresis.

The PCR products of 16S rRNA gene and ITS1-5.8S rDNA -ITS2region were gel-purified and cloned into the pGEM-T vector (TaKaRa) for sequencing. Qualified single clones were selected for sequencing (Ige Biotech Co., Ltd., Guangzhou, China). Ten 16S rRNA gene and ITS1-5.8S rDNA -ITS2 sequences were used for pairwise sequence alignments performed by the BLASTN program.

### Genomic sequencing

Strain endobacterium *A. faecali* SCSIO B001 was cultured for 2–3 days in LB media. Then, genomic DNA was extracted using phenol-chloroform extraction and ethanol precipitation^30^. After detection of genomic DNA concentration by Qubit3.0, the whole-genome shotgun project of *Alcaligenes* sp. SCSIO B001 was performed with Illumina Hiseq and third generation sequencing technologies by constructing two different gDNA libraries (Illumina Pe and PacBio(8–10 kb)) at Biozeron Bio-Pharm Technology Co., Ltd, Shanghai, China. The high quality reads were assembled into bp using SOAP de novo v2.04 (http://soap.genomics.org.cn/) and Celera Assembler 8.0. After gap closing by SOPA Gap Closer v1.12, a complete genome was obtained. The genome of *A. faecalis* SCSIO B001 was annotated through the Prokaryotic Genome Annotation Pipeline on NCBI(2013).

Symbiont F190/B001 was cultured for 4–5 days in trypticase soy broth media supplemented with antibiotics (ciprofloxacin hydrochloride) to inhibit the growth of *A. faecalis* SCSIO B001. Then, genomic DNA was extracted using phenol-chloroform extraction and ethanol precipitation. After detection of genomic DNA concentration by Qubit3.0, and collection of genomic DNA by 1% agarose gel electrophoresis, the whole-genome shotgun project of *Spiromastix* sp. SCSIO F190 was performed using Illumina Hiseq and third generation PacBio sequencing technology by two constructing different gDNA libraries (Illumina Pe and PacBio(10–20 kb)) after the DNA was broken into 300–500 bp segments at Biozeron Bio-pharm Technology Co., Ltd, Shanghai, China. The adapter sequences, bases with phred scores below Q20 and reads shorter than 50 bp were removed. After the reads were filtered and clipped, the high quality reads were assembled bp using SOAP *de novo* v2.04 (http://soap.genomics.org.cn/) and Celera Assembler 8.0. After gap closing by SOPA Gap Closer v1.12, a complete genome was obtained. The genome of *Spiromastix* sp. SCSIO F190 was annotated through the Eukaryotic Genome Annotation Pipeline on NCBI (2013).

### Phylogenetic analysis

For phylogenetic analysis, the full-length of the ITS region (including complete ITS1, 5.8S rDNA gene and ITS2) was amplified with primers ITS1 and ITS4 and 16S rRNA gene was amplified with 27 F and 1492 R. The ITS1-5.8S-ITS2 and 16S rRNA gene sequences were compared with the available in GenBank (http://blast.ncbi.nlm.nih.gov/blast.cgi) by using BLAST to determine the phylogenetic affiliation. After multiple alignments of data by CLUSTAL-X with gaps treated as missing data, the phylogenetic tree was constructed in MEGA v5.0. The tree topology of the neighbor-joining data was evaluated by bootstrap analysis with performing 1,000 replicates^31^.

### Confocal laser scanning microscopy

According to the methods detailed by Partida-Martinez and Sulemankhil^8^, growing mycelia of symbiont F190/B001 (0.5 ml) were transferred to a centrifuge tube containing 0.5 mL 0.85% saline. A 10 µL aliquot of the mixture was placed onto a microscope slide and treated with 0.5 µL Live/Dead BacLight SYTO 9 and an equal amount of propidium iodide (Live/Dead BacLight Bacterial Viability Kit, Molecular Probes-L7007). A 15 min incubation in the dark (RT), ProLong Gold antifade reagent (Molecular Probes–P36935) was employed to enhance fluorescence. The sample was then incubated for another 20 min and analysed using a Leica TCS SP8 AOBS Laser Microscope at 510/520 nm.

### Fluorescent in situ hybridization (FISH)

According to the methods reported with a little modification^16^, growing mycelium of the symbiont F190/B001 was suspended in phosphate buffered saline (PBS) and then were transferred into fresh 4% formaldehyde solution (DEPC water preparation) and incubated for 2 h at 4 °C. After fixation, the sumbiont F190/B001 mycelium was dehydrated in a series of ethanol-PBS solutions beginning with 50, 70, and finally 95% ethanol and embedded in paraffin wax. The paraffin wax was then sliced and incubated for 2 h at 62 °C. Dehydrated mycelia were soaked in xylene for 15 min, xylene I for another 15 min, anhydrous ethanol for 5 min and then anhydrous ethanol II for another 5 min. After soaking, the mycelia were washed with ethanol-PBS solutions beginning with 85%, 75% and finally H_2_O [DEPC-treated]. The mycelium slice was boiled in PBS solution for 10–15 min and was digested by protease K (20 µg/mL) at 37 °C for 30 min. Whereafter, the slices were washed with pure H_2_O once and PBS buffer (3×). The prepared mycelium slice was incubated at 37 °C in hybridization buffer (50% formamide, 100 g/mL ssDNA, 20 ng probe, 50 mmol/L DTT, 5 × Denhardt solution, 5% dextran sulfate, 4× SSC) and after 60 min the hybridization buffer was removed. A 2 µL aliquot of BET42a probe^32^ (10 µM), a universal 23S rRNA gene oligonucleotide probe (5′-GCCTTCCCACTTCGTTT-3′; labeled with 5′-6-carboxy tetramethylrhodamine fluorescein tag; excitation, 515 nm; emission, 530 nm; Integrated DNA Technologies, Inc.) targeted to the genome of bacteria was added and the slices were incubated overnight at 25 °C. The hybridized samples were rinsed in 2× SSC buffer (0.3 M NaCl, 0.03 M sodium citrate; pH 6.9–7.1) for 10 min at 37 °C, then 1 × SSC for 10 min at 37 °C, and finally in 0.5× SSC for 10 min at 25 °C. The sample was then analysed using a Leica TCS SP8 AOBS Laser Microscope.

### Microbial fermentation

The fresh mycelium grown on ISP2 agar medium at 28 °C for 4 days was inoculated into 250 mL Erlenmeyer flask containing 100 mL of potato dextrose broth medium. After 3 days of incubation at 28 °C on rotary shakers at 180 rpm, 15 mL aliquots of liquid culture were transferred into each 1000 mL Erlenmeyer flask containing 200 mL of potato dextrose broth medium, and cultivation was carried out at 28 °C for 7 days.

### Isolation and structure elucidation of spiromarmycin

The filtrate of the fermentation broth (50 L) was extracted with butanone (3 × 50 L). All extracts were then combined and the solvent removed in vacuo to afford a yellow oily residue (25.6 g), which was subjected to column chromatography over silica-gel (SiO_2_; 200-300 mesh; Qingdao Marine Chemical Ltd, Qingdao, China) eluting with CHCl_3_/MeOH mixtures of a progressively increasing polarity (100:0, 98:2, 96:4, 95:5, 90:10, 80:20, and 50:50, v/v) to afford seven primary fractions (F1,1.2 g; F2, 2.6 g; F3, 4.9 g; F3, 1.2 g F4, 1.3 g; F5, 1.3 g; F6, 2.3 g and F7, 0.8 g). Bioassays revealed that fraction F3 (96:4 CHCl_3_/MeOH) showed good antibacterial activity against MRSA. F3 fractionation over silica gel using (96:4 CHCl_3_/MeOH) rendered the active components contained within this primary fraction (356 mg). The principal active component of F3 was purified by semi-preparative HPLC with ODS column using an elution system consisting of solvent A (CH_3_CN) and solvent B (H_2_O), eluting over the duration of 30 min (2.0 mL/min) to yield compounds **1a** and **1b** (16.5, 15.5 mg).

Spiromarmycin **1a** was obtained as orange crystals and its molecular formula C_19_H_24_O_6_ was deduced from HRESIMS (*m/z* 371.1470 [M + Na]^+^, calcd for C_19_H_24_O_6_Na: 371.1471) and ^13^C NMR spectra. The ^1^H NMR (Supplementary Table 4) of **1** indicated the presence of three methyls (*δ*H 0.88, 1.37, and 1.45), five methylene (*δ*H 1.84, 1.42, 1.30, 1.30, 2.63, and 2.88), two methine (*δ*H 1.37, 5.61 and 6.31), one olefinic methines (*δ*H 6.31). The ^13^C NMR spectra (Supplementary Table 4) of **1** showed 19 carbon signals in conjunction with DEPT spectrum indicating the presence of three methyls, five methylenes, one olefinic group, six quaternary olefinic carbons, one oxygenated quaternary carbon, one ester carbonyl, and one enone carbonyl. The structure of the tricyclic dione was confirmed by the observation of the two and three-bond HMBC correlations: H-9 to C-1 (*δ*_C_ 163.1), C-7 (*δ*_C_ 142.4), and C-8 (*δ*_C_ 114.3); H-2 to C-1 (*δ*_C_ 163.1), C-3 (*δ*_C_ 140.3), C-8 (*δ*_C_ 114.3); H-15 to C-3 (*δ*_C_ 140.3), C-4 (*δ*_C_ 114.9), C-5 (*δ*_C_ 146.1), C-16 (*δ*_C_ 68.3), C-17 (*δ*_C_ 79.4); and H-16 to C-4 (*δ*_C_ 114.9). The pentyl moiety of 1 was clarified as follows: a triplet methyl at *δ*H 0.88 (J = 6.5 Hz), four methylene at *δ*H 1.84 (2H, m), 1.42 (2H, m), 1.30 (2H, m), and 1.30 (2H, m) with ^1^H-^1^H COSY correlations of H-14/H-13 and H-11/H-10/H-9 and the ^13^C NMR spectra indicated the presence of two methyls and four methylenes, and HMBC correlations of H-14 to C-12 (*δ*_C_ 31.3) and C-13 (*δ*_C_ 22.5); H-12 to C-13 (*δ*_C_ 22.5); H-11 to C-12 (*δ*_C_ 31.3); H-9 to C-10(*δ*_C_ 36.73); H-10 to C-8 (*δ*_C_ 114.3) thus establishing linkage of the pentyl structure to C-9.The locations of the two methyl groups (*δ*H 1.37, and 1.45) were determined by their HMBC correlations with C-17 (*δ*_C_ 79.4), C-16 (*δ*_C_ 68.3), C-19 (*δ*_C_ 21.9), and C-16 (*δ*_C_ 68.3), C-17 (*δ*_C_ 79.4), C-18(*δ*_C_ 24.4) (see Supplementary Figs. 22–27).

Spiromarmycin species **1b** was obtained also as orange crystals and its molecular formula C_19_H_24_O_6_ was deduced from HRESI-MS (*m/z* 371.1469 [M + Na]^+^, calcd. for C_19_H_24_O_6_Na: 371.1471) and ^13^C NMR spectra. The NMR data of **1b** were very similar to those of originally identified **1** (the mixture of **1a** and **1b**) (see Supplementary Figs. 28–33, Supplementary Table 4).

Spiromarmycin **1a**: orange crystals; $[\alpha ]_{\mathrm{D}}^{25}$ = −77.6 (0.001 M in MeOH); UV/Vis (MeOH): λ_max_ 210.8, 352.2 nm; ^1^H NMR (500 MHz, CDCl_3_) and ^13^C NMR (126 MHz, CDCl_3_), ^1^H and ^13^C NMR data, see Supplementary Table 4; NMR spectra see Supplementary Figs. 22–27. (+)-HRESIMS *m/z* [M + Na]^+^ 371.1470 (calcd for C_19_H_24_O_6_Na: 371.1471), see Supplementary Fig. 6.

Spiromarmycin **1b**: orange crystals; $[\alpha ]_{\mathrm{D}}^{25}$ = +118 (0.001 M in MeOH); UV/Vis: λ_max_ 210.8, 352.2 nm; ^1^H NMR (500 MHz, CDCl_3_) and ^13^C NMR (126 MHz, CDCl_3_); ^1^H and ^13^C NMR data, see Supplementary Table 4; NMR spectra see Supplementary Figs. 28–33.; (+)-HRESIMS *m/z* [M + Na]^+^ 371.1469 (calcd for C_19_H_24_O_6_Na: 371.1471), see Supplementary Fig. 7.

To expore the mechanism for C-9 eperization of compound **1**, **1a** (0.4 mg) was incubated in D_2_O at room temperature. After overnight incubation, the **1a** and D_2_O mixture was dissolved in CDCl_3_ and subsequently subjected to ^1^H NMR spectroscopic analysis. The equal ratio of **1a** and **1b** (0.2 mg of each compound) dissolved with CDCl_3_ was set as a control and also subjected to ^1^H NMR spectroscopic analysis.

### X-ray crystallography of spiromarmycin

Orange crystals of compound **1a** were obtained from a mixture of MeOH: CH_3_COCH_3_:H_2_O (80:15:5). A slice of the applicable crystal was selected to perform single crystal diffraction analysis by single crystal X-ray diffractmeter system with model of XtaLAB PRO MM007HF. The diffractometer was equipped with a Pilatus200K Silicon Diaray Detector and Cu K_*α*_ radiation generator. The crystal was kept at T = 99.99 (10) K during data collection. The ShelXT structure solution program was used to solve the structure with the dual solution method and thereafter full-matrix least squares difference Fourier techniques was used for refinement. Crystallographic data for spiromarmycin have been deposited with the Cambridge Crystallographic Data Center with the deposition number CCDC 2016950. Copies of the data can be obtained, free of charge, on application to the Director, CCDC, 12 Union Road, Cambridge CB2 1EZ, UK [fax: t44(0)-1233-336033 or e-mail: deposit@ccdc.cam.ac.uk].

### Gene expression analysis of *spm* cluster

Symbiont F190/B001 used for RNA extraction were grown in PDA medium with or without the supplemementation of antimicrobial ciprofloxacin hydrochloride for 4 days. Total RNAs was extracted from fresh mycelia using RNAiso Plus reagent (TaKaRa) according to the manufacturer’s instructions. The chromosomal DNA was removed by using RNase-free DNase I (New England Biolabs). Reverse transcription was performed with the Transcriptor First Strand cDNA Synthesis Kit (Roche) using 1 μg total RNA and the anchored-oligo(dT)18 primer. The quntatitatve real time RT-PCR was performed using FastStart Essential DNA Green Master (Roche) by LightCycler 96 Instrument (Roche). The fragments of about 100-150 bp of the targeted genes, ITS1, *spm12* and *spm13*, were amplified with primers ITS1-qPCR-F (5′-TGGTGAATTGAGCGGTCTAAG-3′)/ITS1-qPCR-R (5′-ACTTGAGCGGGTGATAACG-3′), spm12-qPCR-F (5′-GCAGCGTATATCGTCAATGTTC-3′)/spm12-qPCR-R (5′- TCTCTTCGTGGCAGTCTCG-3′), and spm13-qPCR-F (5′-CCAGGCGCAGTATGCTGCT-3′)/spm13-qPCR-R (5′- CATGCCTCGTCATCGCGCT-3′), respectively. The relative mRNA level of *spm12* and *spm13* were normalized to that of ITS1.

### Biosynthetic studies

Stable isotope label feeding experiments with *Spiromastix* sp. SCSIO F190 were performed according to the protocol used for the production and isolation of spiromarmycin with little modification^33^. After inoculation of the symbiont pairing, sodium [1-^13^C] acetate (200 mg/200 mL of media × 5 flasks) was filtered through millipore filters (0.2 μm) and was added in thirds at 24, 32, and 40 h. Then, the labeled products spiromarmycin **1a** (8.5 mg) and spiromarmycin **1b** (12.5 mg) were isolated from fractionation of a 20 d culture. A similar feeding experiment of sodium [2-^13^C] acetate provided the labeled spiromarmycin **1a** (12.6 mg), and spiromarmycin **1b** (13.3 mg), respectively. Supplementary Fig. 11 indicates isotopic enrichment which supports a PKS-medoated assembly of spiromarmycin.

### Effects of B001 on metabolic profile of symbiont F190/B001

Symbiont F190/B001 were first grown on ISP2 medium at 28 °C for 5–7 days to achieve sporulation and then inoculated into a 250 mL erlenmeyer flask containing 50 mL of potato dextrose broth medium followed by culturing for 4 days at 200 rpm and 28 °C. 1 mL aliquots of this 4-days culture were transferred as seed into each 250 mL Erlenmeyer flask containing 50 mL of potato dextrose broth medium supplemented with antimicrobial at the concentration of 100 μg/mL (ampicillin, kanamycin, chloromycetin, or ciprofloxacin hydrochloride); cultivation was carried out at 200 rpm and 28 °C for 7 days. The culture filtrate (300 mL) was extracted with butanone (1 × 50 mL). All extracts were redissolved 200 μL of MeOH and centrifuged at 12,000 × *g* for 10 min. High-performance liquid chromatography (HPLC) analyses were performed on an Agilent 1260 HPLC (Agilent Technologies, Santa Clara, CA, United States) equipped with a Phenomenex ODS column (150 × 4.6 mm, 5 μm) with a linear gradient of 0–100% solvent B (solvent B: 0.1% HOAc-85% CH_3_CN in H_2_O; solvent A: 0.1% HOAc-15% CH_3_CN in H_2_O) over the course of 30 min at a flow rate of 1 mL/min; detection was carried out at 254 nm.

### GFP-Labeled *A. faecalis* SCSIO B001

GFP-Labeled endobacterium *A. faecalis* SCSIO B001 was constructed using plasmid pHGEI01^34^, which is an integrative plasmid and used to insert exogenous gene fragment into the desired locus at the chromosome. First, the RNA polymerase α subunit gene promoter (named *RpoA*p) was amplified from *A. faecali* SCSIO B001 genomic DNA with primers RpoA- pHGEI01-5′-O: (5′-CGTATAATGTATGCTATACGAACGGTAttttgtacgcgaagacctcttacgc-3′; the uppercase letter represents the homologous sequence on the pHGEI01 vector sequence and the lowercase letter represents the homologous sequence of the *RpoA*p sequence) and RpoA-GFP-3′-I: (5′-CGACGGCCAGTGCCAAGCTTGTCGACttatttgtatagttcatccatgccatgtg-3′; the uppercase letter represents the homologous sequence of GFP gene and the lowercase letter represents the homologous sequence of the *RpoA*p sequence). Amplification was accomplished in a 50 µL reaction mixture containing 1 µL of genomic DNA (~50 ng), 0.5 µL of FastPfu DNA Polymerase (1 U) (Transgen Biotechnology Co., Ltd., Beijing, China), 5 µL of reaction buffer (10×) and 5 µL of dNTPs and 1 µL of each primer, and 36.5 µL of sterile Milli-Q water. The PCR conditions called for 30 cycles at 95 °C for 45 s, 58 °C for 45 s, and 72 °C for 30 s and 10 min at 72 °C for the final chain elongation. PCR products of the expected size were purified (153 bp) by application of the E.Z.N.A. Gel Extraction Kit (Omega Bio-tek, Inc) according to the manufacturer’s instructions.

The GFP gene was then amplified from plasmid pHGM01-MCP::GFP^35^ with primers GFP-RpoA-5′-I: (5′-CGAGGGTATTGAAAAGGAAATCGAatgagaggatctcaccatcaccatc-3′; the uppercase letter represents the homologous sequence of *RpoA*p sequence and the lowercase letter represents the homologous sequence of the GFP gene sequence) and GFP- pHGEI01-3′-O: (5′-CGACGGCCAGTGCCAAGCTTGTCGACttatttgtatagttcatccatgcc-3′; the uppercase letter represents the homologous sequence the homologous sequence on the pHGEI01 vector sequence and the lowercase letter represents the homologous sequence of the GFP gene sequence). Amplification was accomplished in a 50 µL reaction mixture containing 0.5 µL of genomic DNA (~50 ng), 0.5 μL Primerstar DNA polymerase (TaKaRa Biotechnology (Dalian) Co., Ltd.), 25 μL reaction buffer (2×), 2.0 µL of each primer, 5 μL of dNTPs (2.5 mM each) and 15.0 μL of sterile Milli-Q water. The PCR conditions were 30 cycles at 95 °C for 30 s, 58 °C for 30 s, and 72 °C for 45 s and 10 min at 72 °C for a final chain elongation. PCR products of the expected size (780 bp) were purified using the E.Z.N.A. Gel Extraction Kit (Omega Bio-tek, Inc) according to the manufacturer’s instructions.

The *RpoA*p and GFP gene fragments were then joined together by fusion PCR method to obtain product of *RpoA*p-GFP (933 bp). Amplification was accomplished in a 50 µL reaction mixture containing 0.25 µL of the purified PCR products of *RpoA*p (~1.0 ng), 0.25 µL of the purified PCR products of GFP gene (~1.0 ng), 0.5 μL Primerstar DNA polymerase (TaKaRa Biotechnology (Dalian) Co., Ltd.), 25 μL reaction buffer (2×), 2.0 µL of primer RpoA-pHGEI01-5′-O, 2.0 µL of primer GFP-pHGEI01-3′-O, 5 μL of dNTPs (2.5 mM each) and 15.0 μL of sterile Milli-Q water. The PCR conditions called for two steps. First step: 7 cycles at 95 °C for 30 s, 58 °C for 30 s, and 72 °C for 60 s and 10 min at 72 °C for the final chain elongation. Second step: 25 cycles at 95 °C for 30 s, 62 °C for 30 s, and 72 °C for 60 s and 10 min at 72 °C for the final chain elongation. PCR products of the expected size (933 bp) were obtained using the E.Z.N.A. Gel Extraction Kit (Omega Bio-tek, Inc) according to manufacturer’s instructions.

The pHGEI01 vector was linearized by *Bam*HI/*Eco*RI digestion. The products of the expected size (~6.9 Kb) were purified by E.Z.N.A. Gel Extraction Kit (Omega Bio-tek, Inc) according to the manufacturer’s instructions. Then, the *RpoA*p-GFP fragment was inserted into linearized pHGEI01 vector—using the the Vazyme ClonExpress II One Step Cloning Kit (Vazyme, Nanjing, China) to yield recombinant plasmid pHGEI01-*RpoA*p-GFP. The constructed vector was transformed into *E. coli* WM3064 (a dap auxotroph, derivative of strain β2155); the GFP with RNA polymerase α promoter was integrated into the genome of endobacterium *A. faecalis* SCSIO B001 following conjugational transfer. The monoclonal GFP-labeled *A. faecalis* SCSIO B001 was purified from LB medium plate containing antibiotic kanamycin.

### Reinfection assay

The reinfection assay was performed as previously described but with slight modification^10^. The symbiont F190/B001 was grown for 5–7 days on ISP2 medium plates. GFP-labeled *A. faecalis* SCSIO B001 was first grown in liquid media (LB with kanamycin) for 2 days. Then, 200 μL of bacterial culture was plated onto the fungal petri dish and cocultivation was carried out for 1 week. The sample was then washed with fresh saline (3 X) and the remaining symbiont visualized using a Leica TCS SP8 AOBS Laser Microscope at 510/520 nm.

### Antibacterial assays

Spiromarmycin was dissolved in MeOH at a concentration of 6 mg/mL and appropriate media to be used for each organism to be evaluated were sprayed with suspensions of each organism (including a panel of 76 bacterial targets). Having established appropriate cultures for each organism, 5 μL spiromarmycin soaked into 6 mm diameter sterile filter disks, was applied to each microorganism-bearing medium^36^. Organisms were incubated for 16 h at 37 °C, and diameters of zones of inhibition were measured (in mm) for each plate. Ampicycin, kanamycin, ciprofloxacin, polymyxin B, amphotericin and fluconazole served as positive controls for all relevant microorganisms evaluated.

Minimum inhibitory concentration values were determined using a 96-well plate format with MH broth, as previously described^37^. Briefly, spiromarmycin (**1**) was first dissolved in DMSO at a concentration of 3.2 mg/mL and 2 μL of this stock solution was serially diluted into 98 μL of MH broth. Then, sequential twofold serial dilutions of the solution containing spiromarmycin was serially diluted into 50 μL MH broth and 50 μL cell cultures were added to wells. MIC values of 76 bacterial targets, performed in duplicate, were then determined after incubation at 37 °C for 16–18 h. Ampicycin, kanamycin, ciprofloxacin, polymyxin B, amphotericin, and fluconazole served as positive controls, respectively.

### Antifungal assays

Antifungal assays were performed as previously described but with slight modification^36^. Purified sample was dissolved in a volume of acetone to obtain the final concentration of 1 mg/mL spiromarmycin. Twofold serial dilutions were then carried out to obtain less concentrated stock solutions and these were then mixed completely with liquid sterilized malt extracts agar medium in Petri dishes (9 cm diameter). Following the transfer of three mycelium discs (each 5 mm in diameter) of phytopathogenic fungi onto the solidified media, testing plates were incubated at 28 ± 1 °C for 4–5 days. Cycloheximide was used as a positive antifungal control and used for comparative purposes whereas acetone solvent was used as blank control samples. Antifungal indices were calculated as follows: Antifungal index (%) = (1−Da/Db) × 100. In the context of the antifungal index calculations, Da was the diameter of growth zone in the experimental group dish (mm) and Db was the diameter of the control group dish (mm). The IC_50_ values (the concentration of spiromarmycin that inhibited 50% of the mycelial growth) were calculated by probit analysis.

### Statistics and reproducibility

Antifungal experiments were performed in triplicate and data are showed as mean values ± standard deviation (SD). For the minimum inhibitory concentration values of the antibacterial activities, experiments were performed intriplicate independently using 96-well pates. For transcriptional analysis were performed in triplicate and data represented as means ± SD (*n* = 3); *p* < 0.05 (statistical analysis was performed in Microsoft Excel by Student’s *t* test).

### Reporting summary

Further information on research design is available in the Nature Research Reporting Summary linked to this article.

## Supplementary information

Supplementary Information

Description of Additional Supplementary Files

Supplementary Data 1

Reporting summary

## Data Availability

The genomes of *A. faecalis* SCSIO B001 and *Spiromastix* sp. F190 have been submitted to the NCBI and are covered by the NCBI BioProject numbers PRJNA598809 and PRJNA598808, respectively. Crystallographic data for spiromarmycin have been deposited with the Cambridge Crystallographic Data Center with the deposition number CCDC 2016950. Source data underlying the graphs in figures have been provided in Supplementary Data 1. The authors declare that all other relevant data supporting the findings of this study are available within the article and its Supplementary files, or from the corresponding authors upon request.
