# Supplementary material for: Upregulation of a marine fungal biosynthetic gene cluster by an endobacterial symbiont
Source: Commun Biol. 2020 Sep 23;3:527. doi: 10.1038/s42003-020-01239-y (PMC7511336; doi:10.1038/s42003-020-01239-y)
Supplement: Supplementary file 1 — Supplementary Information [file 42003_2020_1239_MOESM1_ESM.pdf]

Supplementary Information

Upregulation of a Marine Fungal Biosynthetic Gene Cluster by an Endobacterial Symbiont

Mingwei Shao[1,2,3], Changli Sun[1,3], Xiaoxiao Liu[1,3], Xiaoxue Wang[1,2,3], Wenli Li,[4,5] Xiaoyi Wei[6], Qinglian Li[1,3*] and Jianhua Ju[1,2,3*]

[1]CAS Key Laboratory of Tropical Marine Bio-resources and Ecology, Guangdong Key Laboratory of Marine Materia Medica, RNAM Center for Marine Microbiology, South China Sea Institute of Oceanology, Chinese Academy of Sciences, 164 West Xingang Road, Guangzhou 510301, China. [2]College of Oceanology, University of Chinese Academy of Sciences, Beijing 100049, China. [3]Southern Marine Science and Engineering Guangdong Laboratory (Guangzhou), Guangzhou 511458, China. [4]Key Laboratory of Marine Drugs, Ministry of Education of China, School of Medicine and Pharmacy, Ocean University of China, Qingdao 266003, China. [5]Laboratory for Marine Drugs and Bioproducts, Qingdao National Laboratory for Marine Science and Technology, Qingdao 266237, China. [6]Key Laboratory of Plant Conservation and Sustainable Utilization, South China Botanical Garden, Chinese Academy of Sciences, Guangzhou 510650, China.

To whom correspondence should be addressed.

*e-mail: liql@scsio.ac.cn; jju@scsio.ac.c

Supplementary Figures

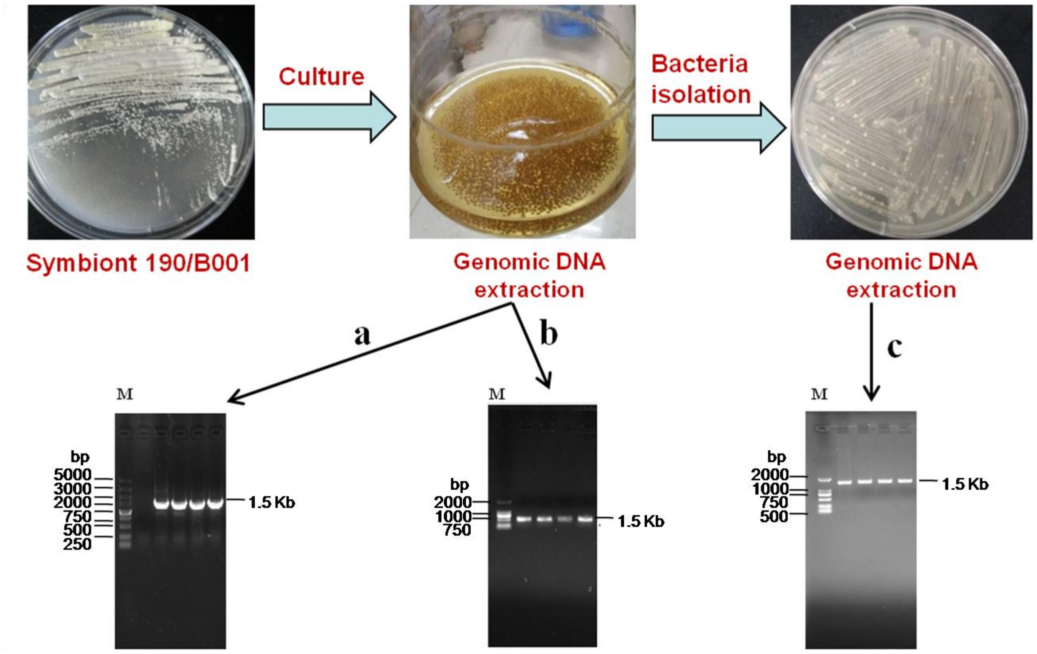

Supplementary Fig. 1. Identification of fungal-bacterial symbiont pair F190/B001. (a) PCR validation was performed on bacterial 16S rRNA gene primer 27F, 1492R fungal genome as template and a clear 1.5 Kb size band was visualized, which was identified as *A. faecali* by sequencing results. (b) PCR validation was performed on fungal ITS1-5.8S rDNA-ITS2 primer ITS1 and ITS4. The sequencing results indicate that *Spiromastix* sp. SCSIO F190 may be a potential new fungal species. (c) With 27F, 1492R as primers for PCR to purified bacterial genome as template, a clear 1.5 Kb size stripe can be obtained. The purified bacteria was identified as *A. faecali* SCSIO B001 by sequencing results, which was same as 16S rRNA gene sequence obtained from total symbiotic genome DNA. See Supplementary Fig. 34 for all the uncut and full gels.

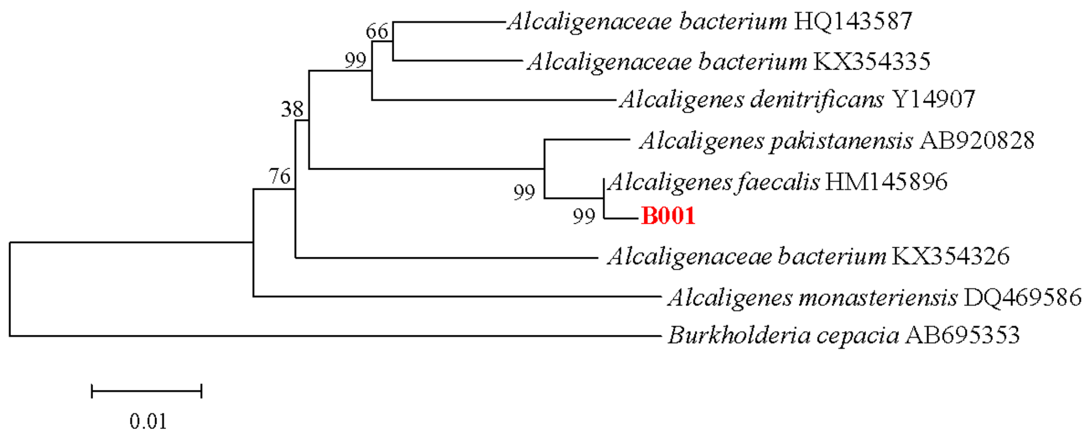

Supplementary Fig. 2. Neighbor-joining phylogenetic tree of 16S rDNA sequences of *A. faecali* SCSIO B001. Bootstrap values were calculated using 1,000 replicates.

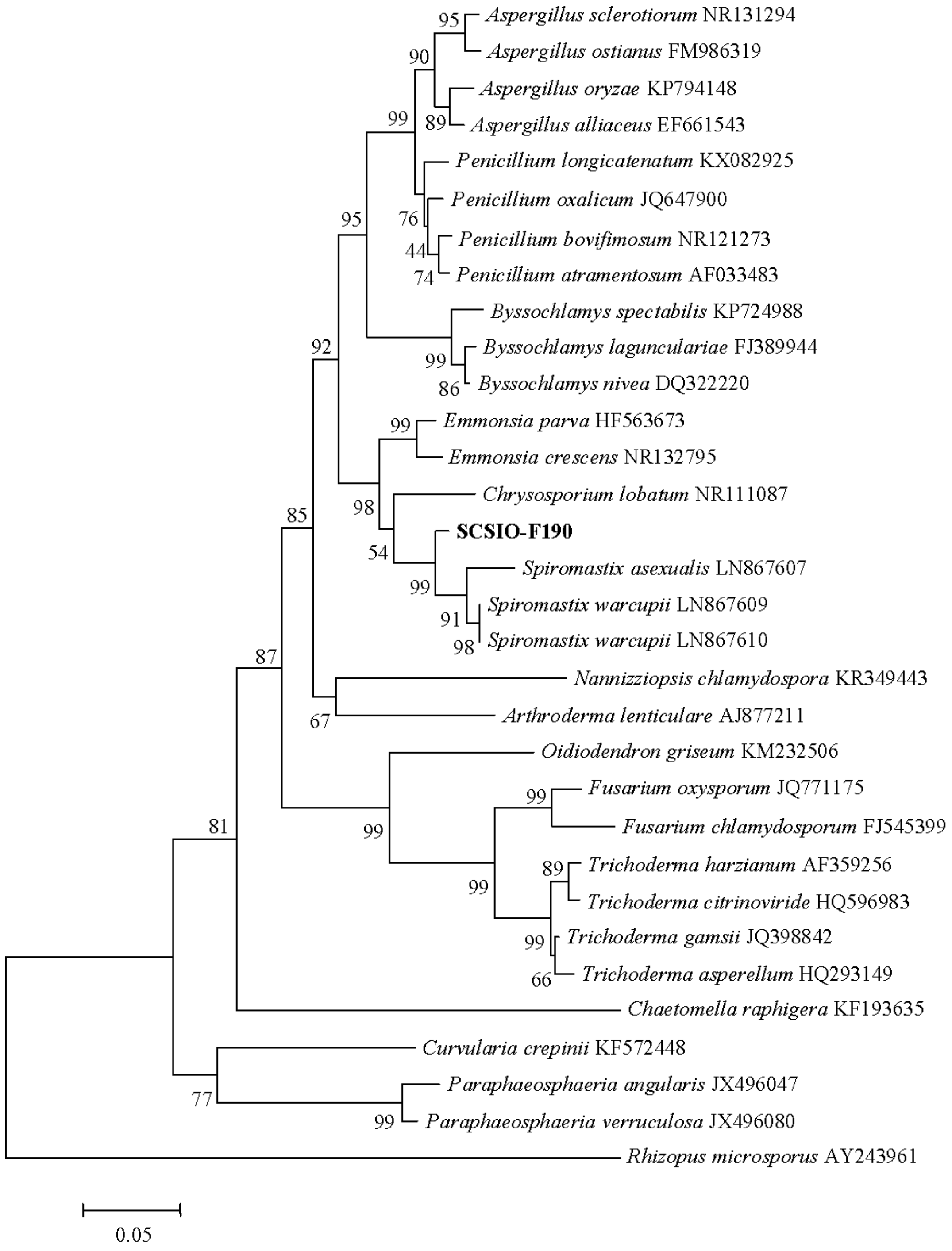

Supplementary Fig. 3. Neighbor-joining phylogenetic tree of ITS1-5.8S-ITS2 rDNA sequences of symbiont F190/B001 isolates. Bootstrap values were calculated using 1,000 replicates.

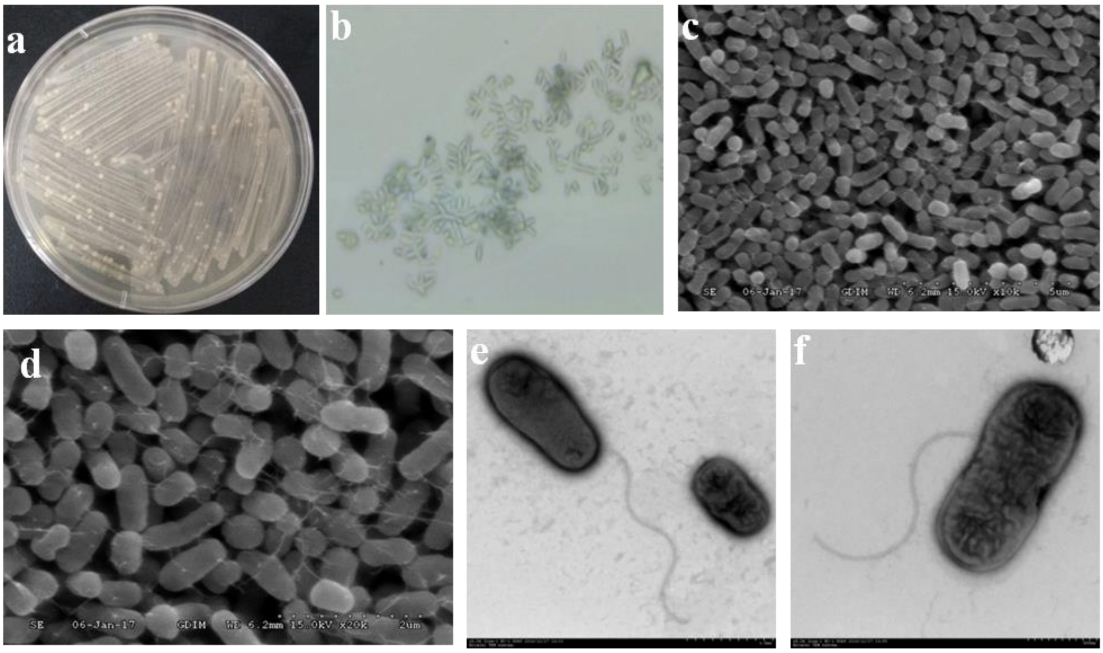

Supplementary Fig. 4. Morphological identification of endobacterium *A. faecali* SCSIO B001. (a) The morphology of the endobacterium *A. faecali* SCSIO B001 on the Luria-Bertani (LB) medium plate. (b) The morphology of endobacterium *A. faecali* SCSIO B001 under optical microscope (400*). (c–d) The morphology of endobacterium *A. faecali* SCSIO B001 under electron scanning microscope. (e–f) The transmission electron microscopy of endobacterium *A. faecali* SCSIO B001 shows apparent flagella.

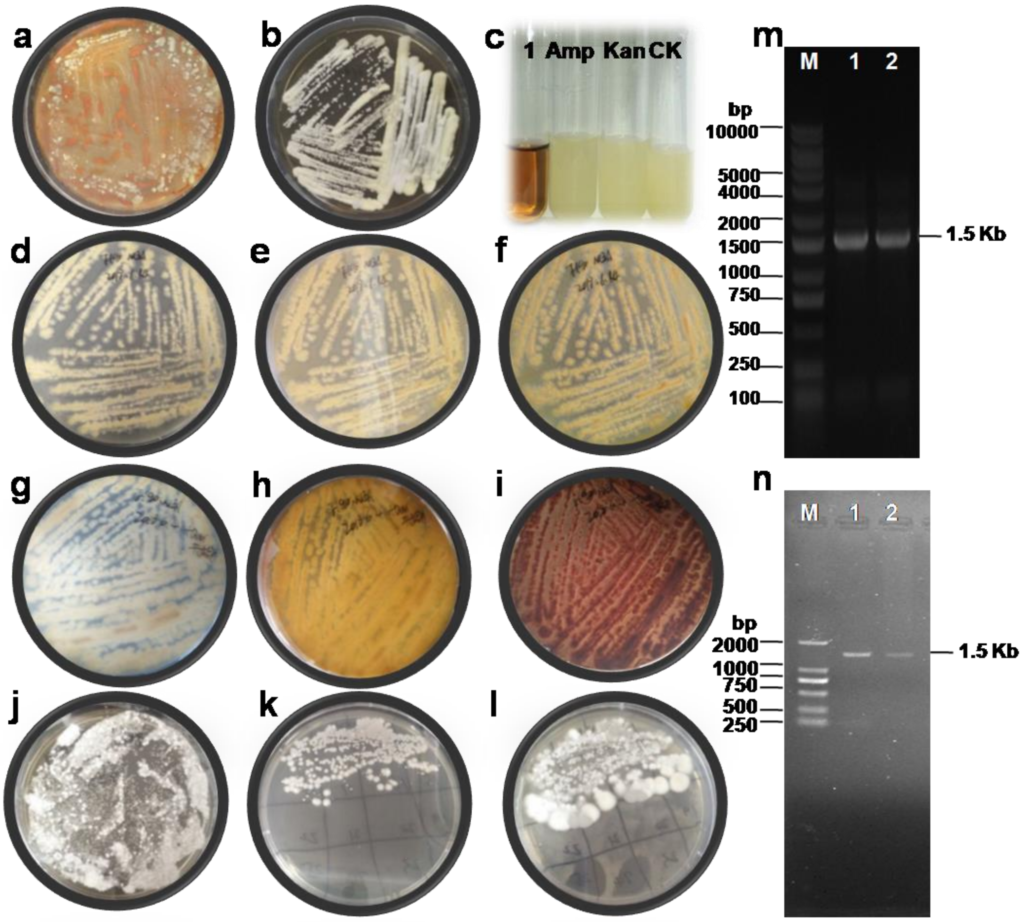

Supplementary Fig. 5. Morphological characterization of the symbiont F190/B001 untreated/treated with antibiotics, grown on solid ISP2 medium for one month, and resistance experiments with spiromarmycin. (a) The morphological characterization of the symbiont F190/B001 untreated with antibiotics. The irregular brown plaques that appear to precipitate out from symbiont F190/B001 were identified as spiromarmycin (**1**). (b) F190/B001 shows fungal morphology when treated with antibiotics (ciprofloxacin hydrochloride). (c) Resistance experiment of spiromarmycin (5*MIC, 80 μg/mL) showed good antibacterial activity against clinical isolates of MRSA without detectable resistance compared with ampicillin (Amp: 80 μg/mL), kanamycin (Kan: 80 μg/mL), and blank control. (d–f) Morphology of the symbiont F190/B001 grown on solid ISP2 medium with antibiotics (ciprofloxacin hydrochloride) for 0.5 month, 1 month and 2 months, respectively. (g–i) Morphology of F190/B001 grown on solid ISP2 medium with antibiotics (ciprofloxacin hydrochloride) and co-cultured with *A. faecali* SCSIO B001 for 0.5 month, 1 month and 2 months, respectively. (j) Morphology of protoplast regenerated F190/B001. (k, l) Morphology of sole clones selected from the regeneration of protoplast using symbiont F190/B001. (m) Symbiont F190/B001 was first treated with different antimicrobial agents, and then the total genomic DNA was extracted and used as template for PCR using 27F, 1492R as primers. A clear 1.5 Kb size stripe of bacterial 16S rRNA gene fragment was clearly obtained. (n) Symbiont F190/B001 was first subjected to protoplast regeneration, and then the total genomic DNA was extracted and used as template for PCR using 27F, 1492R as primers. A clear

1.5 Kb size stripe of bacterial 16S rRNA gene was clearly obtained. See Supplementary Fig. 34 for all the uncut and full gels.

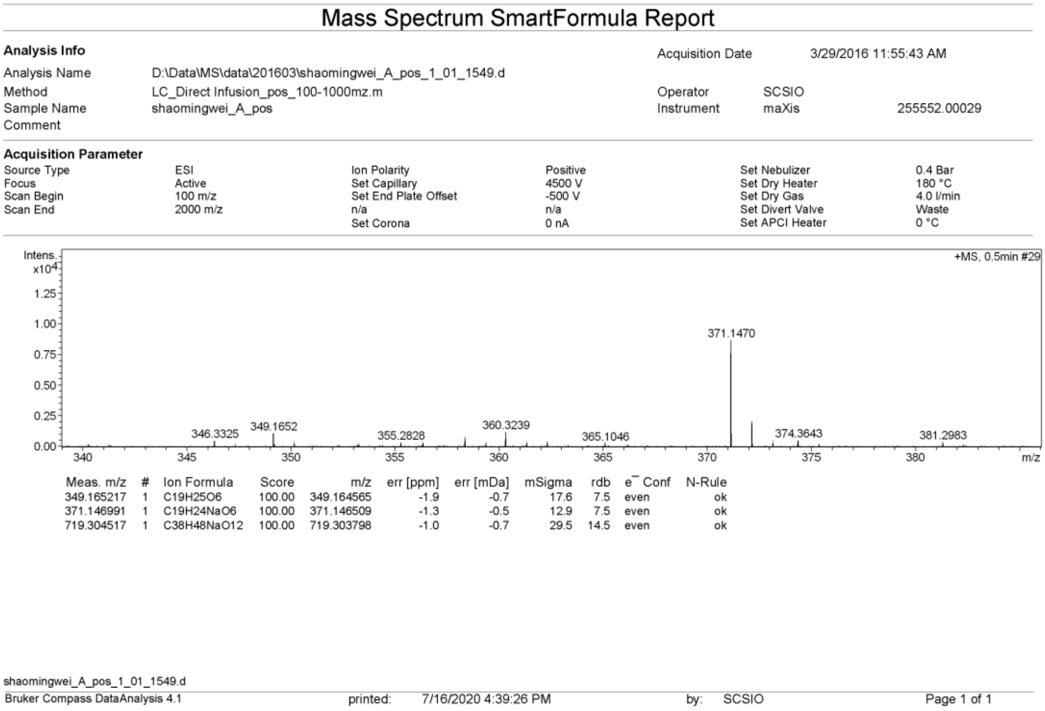

Meas. m/z	#	Ion Formula	Score	m/z	err [ppm]	err [mDa]	mSigma	rdb	e⁻ Conf	N-Rule
349.165217	1	C19H25O6	100.00	349.164565	-1.9	-0.7	17.6	7.5	even	ok
371.146991	1	C19H24NaO6	100.00	371.146509	-1.3	-0.5	12.9	7.5	even	ok
719.304517	1	C38H48NaO12	100.00	719.303798	-1.0	-0.7	29.5	14.5	even	ok

Supplementary Fig. 6. The HRESIMS spectrum of spiromarmycin **1a.**

Mass Spectrum SmartFormula Report

Analysis Info

Analysis Name	D:\Data\MS\data\201603\shaomingwei_B_pos_2_01_1550.d	Acquisition Date	3/29/2016 11:59:04 AM
Method	LC_Direct Infusion_pos_100-1000mz.m	Operator	SCSIO
Sample Name	shaomingwei_B_pos	Instrument	maXis 255552.00029
Comment			

Acquisition Parameter

Source Type	ESI	Ion Polarity	Positive	Set Nebulizer	0.4 Bar
Focus	Active	Set Capillary	4500 V	Set Dry Heater	180 °C
Scan Begin	100 m/z	Set End Plate Offset	-500 V	Set Dry Gas	4.0 l/min
Scan End	2000 m/z	n/a	n/a	Set Divert Valve	Waste
		Set Corona	0 nA	Set APCI Heater	0 °C

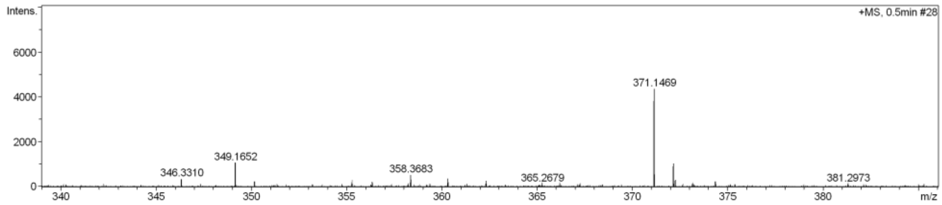

Meas. m/z	#	Ion Formula	Score	m/z	err [ppm]	err [mDa]	mSigma	rdb	e⁻ Conf	N-Rule
349.165157	1	C19H25O6	100.00	349.164565	-1.7	-0.6	9.8	7.5	even	ok
371.146941	1	C19H24NaO6	100.00	371.146509	1.2	0.4	12.6	7.5	even	ok

shaomingwei_B_pos_2_01_1550.d
Bruker Compass DataAnalysis 4.1 printed: 7/16/2020 4:40:03 PM by: SCSIO Page 1 of 1

Supplementary Fig. 7. The HRESIMS spectrum of spiromarmycin **1b**.

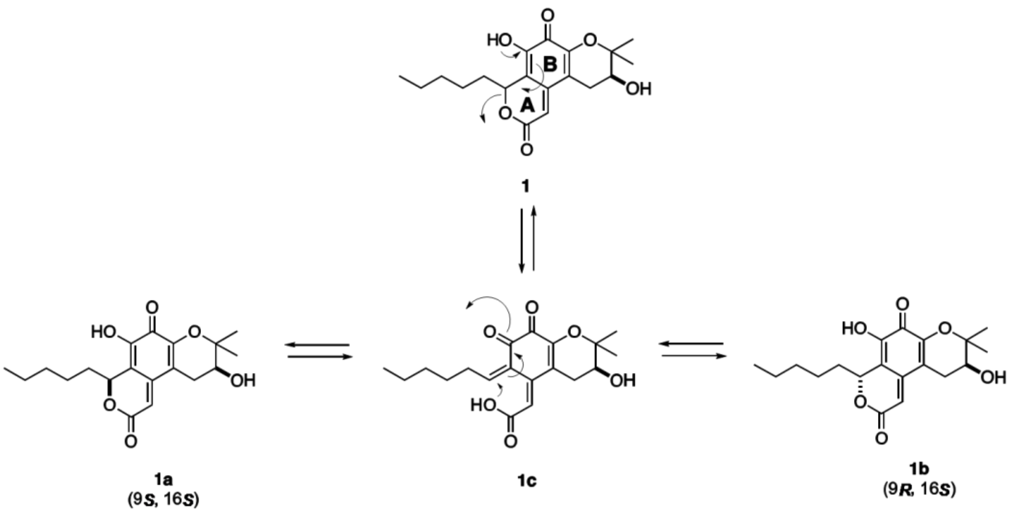

Supplementary Fig. 8. Proposed Mechanism for C9 epimerization and interconversion of 1a and 1b. The allylic nature of C-9, its linkage to the cyclic O of the lactone moiety (ring A) and its conjugation with the α-quinol of ring A appear to activate this position towards racemization starting from either form **1a** or **1b**. We envision a mechanism of C-9 epimerization wherein tautomerization of the enol of ring B, cleaves the C-9 to lactone O bond. The transient acid intermediate **1c** can then recyclize by attacking either face of the transiently planar C-9 enone thus affording either **1a** or **1b** as products. We envision that, given a proton source (MeOH, H_2O, wet DMSO, etc.) this chemistry proceeds quite efficiently but that, in the absence of protons, both **1a** and **1b** are relatively stable; this logic is based on empirical observations during the study of the F190/B001 symbiont pair and its assembly of spiromarmycin.

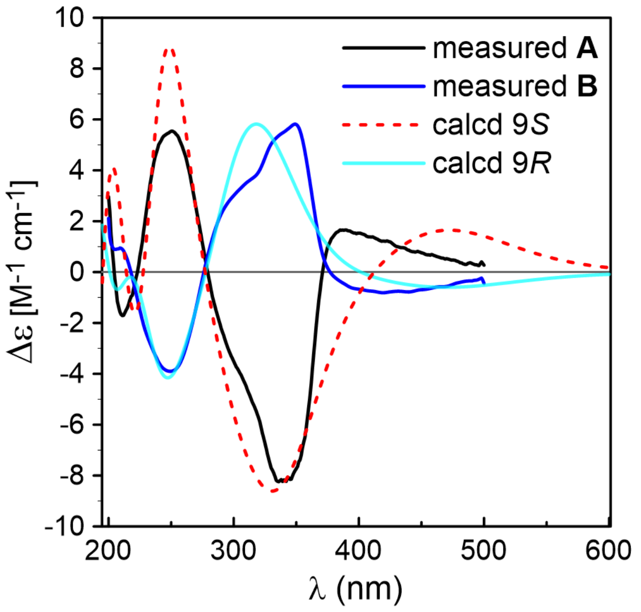

Supplementary Fig. 9. Comparison of the M06-2X/TZVP/PCM calculated ECD spectra of 9*S*, 16*S*-**1** and 9*R*, 16*S*-**1** with the experimental spectra of **1a** and **1b** in MeOH.

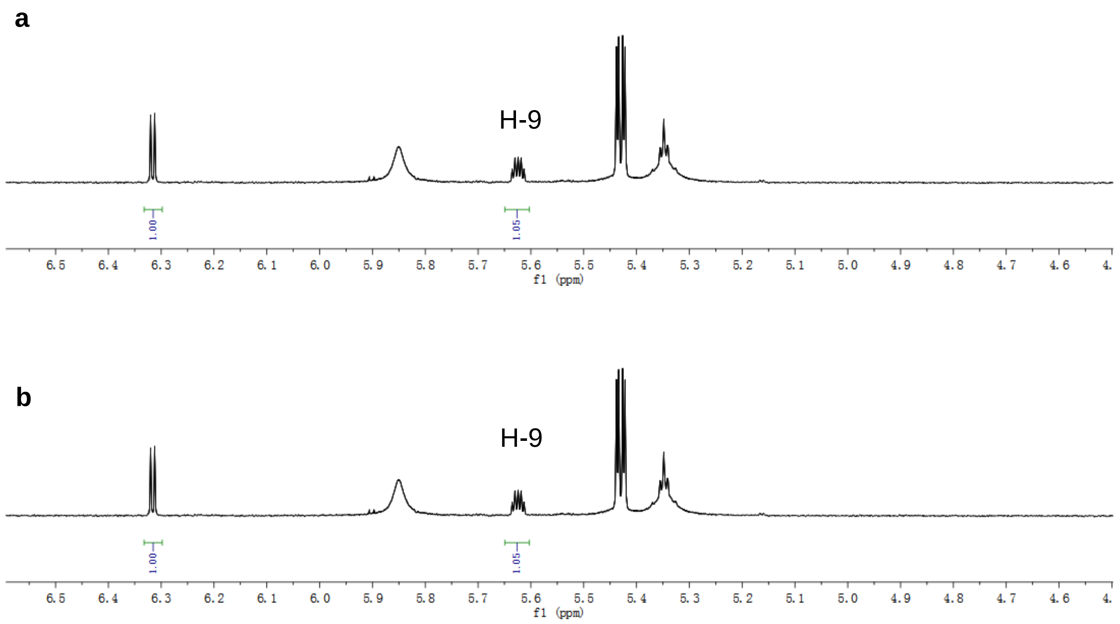

Supplementary Fig. 10. ^{1}H NMR spectrum of D_2O-incubated 1a in $CDCl_3$. (a) The ^{1}H NMR spectrum of equal ratio of **1a** and **1b** (1.2 mg of each compound) in $CDCl_3$. (b) The ^{1}H NMR spectrum of **1a** (2.4 mg) incubated in D_2O at room temperature overnight in $CDCl_3$. No obvious integration change for the C-9 hydrogen was observed.

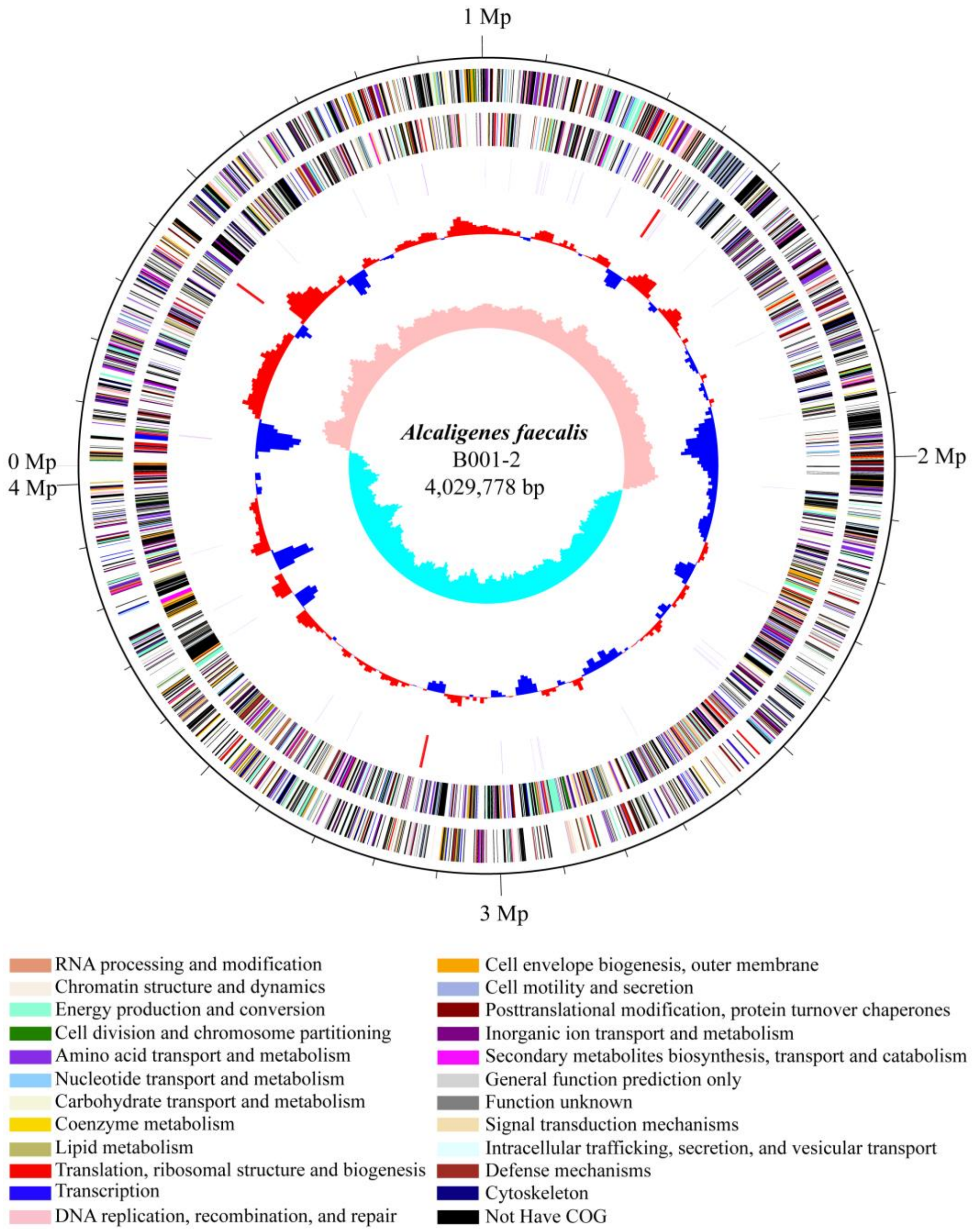

Supplementary Fig. 11. Graphical circular map of *A. faecali* SCSIO B001 genome. Note: The outermost circle identifies genome size, each scale is 0.5 mb; The second and third circles (moving inward) are CDS located on the positive and negative chains, and different colors denote the functional classification of the COG; The fourth circle is rRNA and tRNA; The fifth circle graph is GC content. Red indicates that the GC content of this region is higher than that of the whole genome, and the higher the peak, the greater the difference in average GC content. Blue indicates that the GC content in this region is lower than that of the whole genome, and the higher the peak value, the greater the difference of the average GC content. The inner circle is the GC skew value with the specific algorithm of G-C/G+C. When the value is positive, the positive chain is more prone to transcribing CDS and negative chains are more prone to transcribing CDS, in reverse.

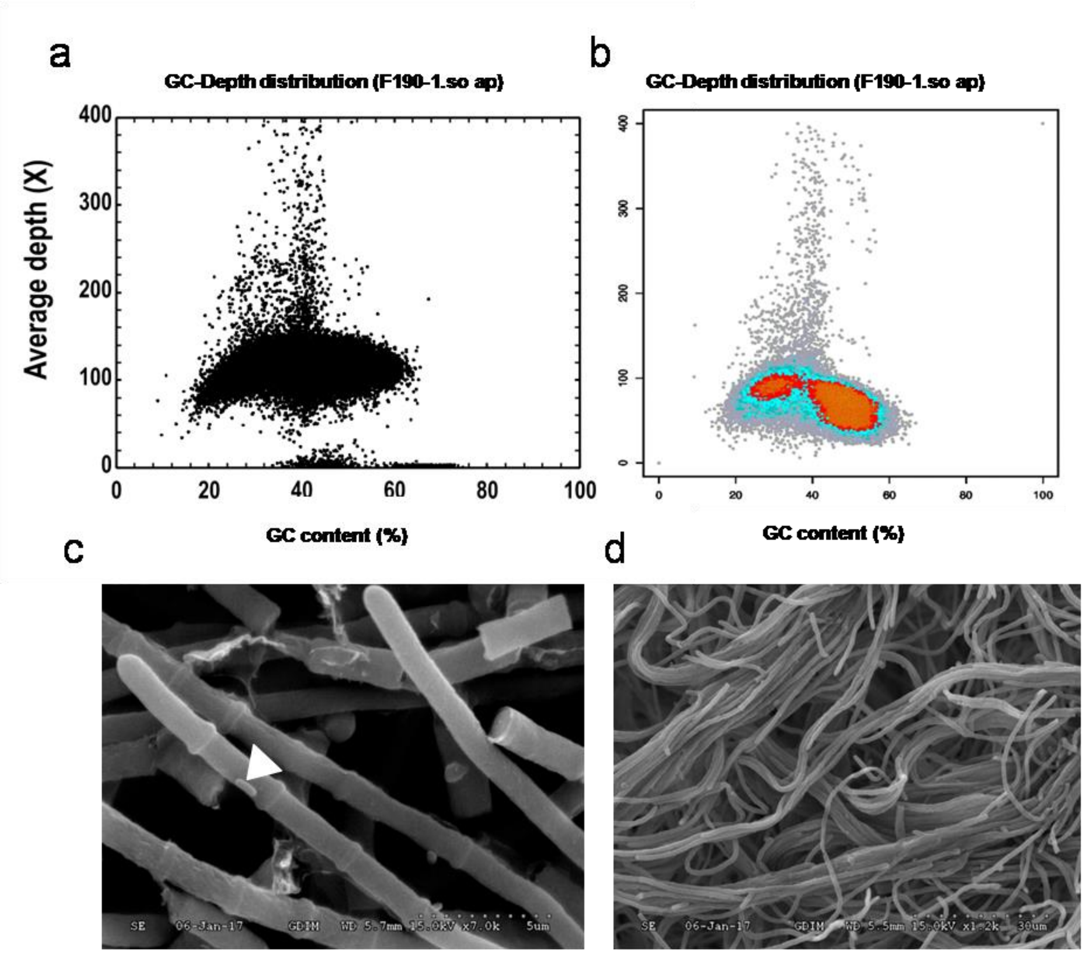

Supplementary Fig. 12. Analysis chart correlating GC content, sequencing depth of *Spiromastix* sp. SCSIO F190 genome and the morphology of F190/B001 symbiont treated with or without antimicrobials under electron scanning microscope. (a) The first genomic sequencing results of symbiont F190/B001 indicates the fungal genome consist of two different species. (b) The second genomic sequencing results of symbiont F190/B001 treated with antibiotics (ciprofloxacin hydrochloride) shows the GC contents became much clearer than the first sequencing results, which indicates that the bacteria attached to the surface of the symbiont F190/B001 can be killed by antibiotics. (c) The morphology of symbiont F190/B001 untreated with antimicrobials under electron scanning microscope. The bacterium attached to the surface of the fungal hyphae is indicated with white spike. (d) The morphology of F190/B001 symbiont treated with antimicrobial ciprofloxacin hydrochloride under electron scanning microscope. No bacteria were observed on the surface of the fugal hyphae.

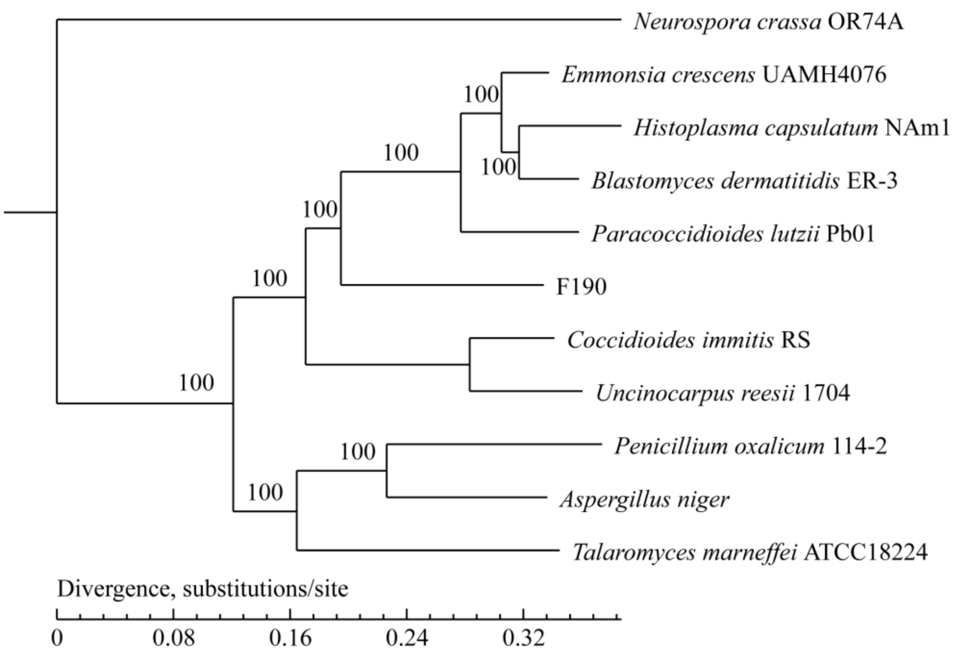

Supplementary Fig. 13. Phylogenetic tree of genomic sequences for F190/B001 isolates. The number on the branch indicates the credibility of the branch, and the closer the value is to 100, the higher credibility. Branch length represents the size of the evolutionary distance, which is calculated by the average number of nucleotide substitutions.

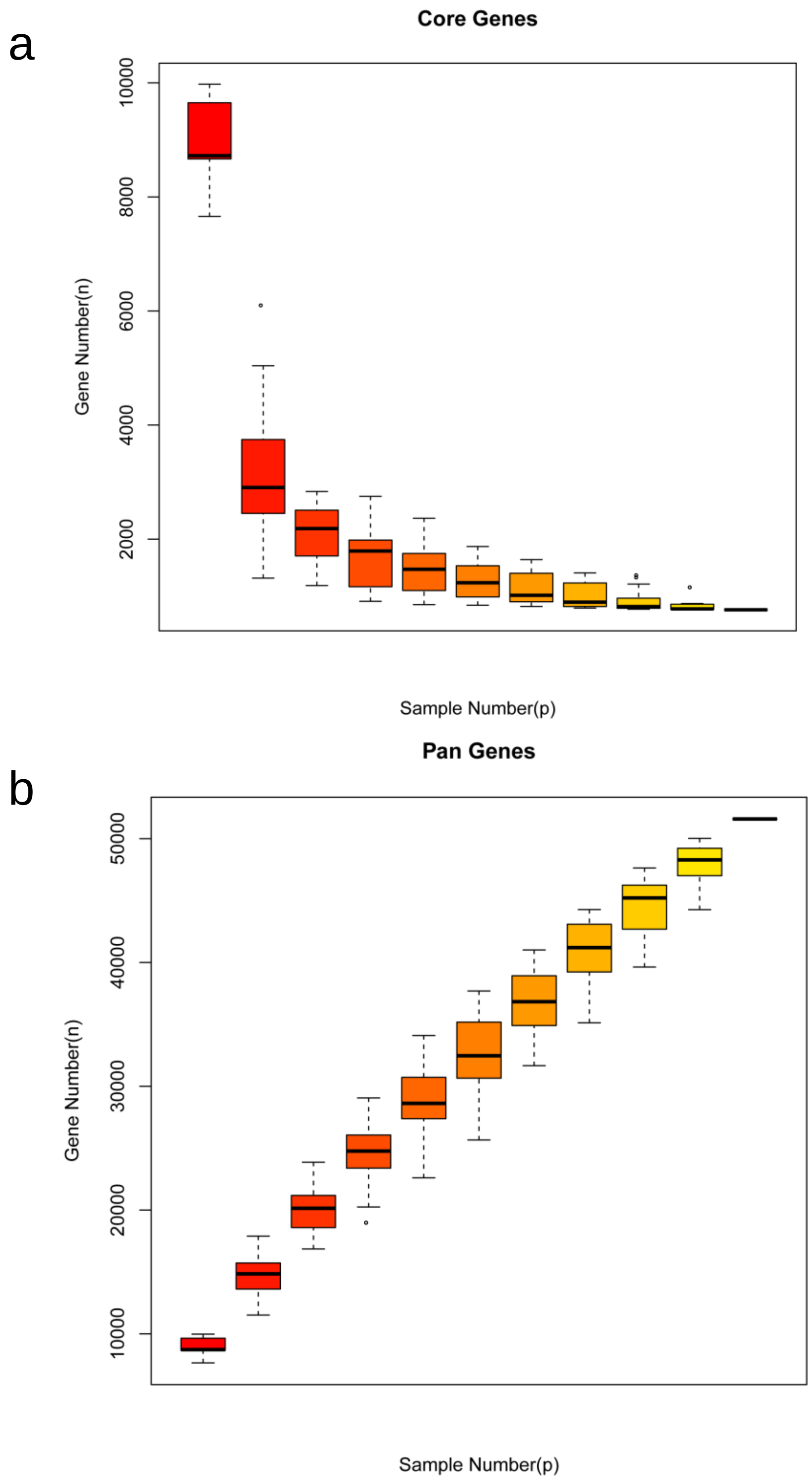

Supplementary Fig. 14. Dilution curve Core-Pan gene of *Spiromastix* sp. SCSIO F190 through the comparison of the protein sequences with selected ten strains. (a) Dilution curve for Core genes; (b) Dilution curve of Pan genes The abscissa represents the number of samples selected for each statistic, and the ordinate represents the distribution of the number of genes.

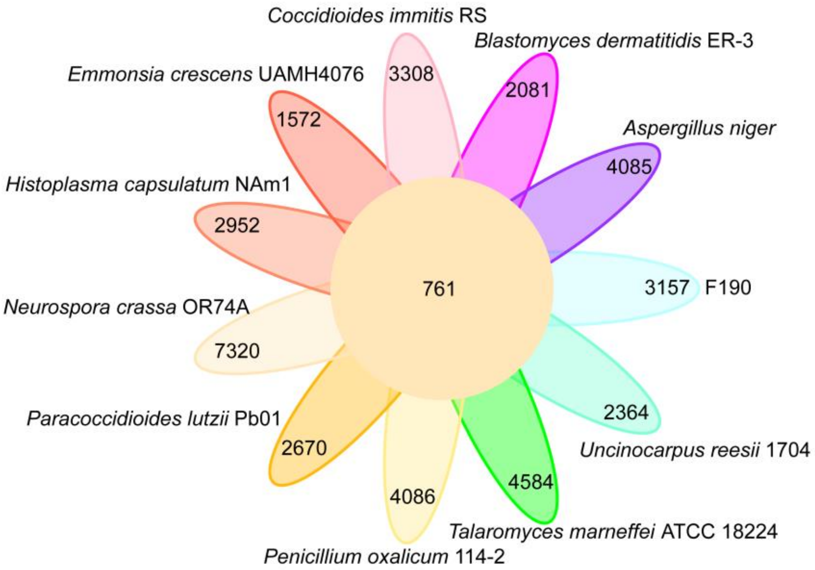

Supplementary Fig. 15. Venn diagram of the Core-Pan gene. Each oval represents a sample, the number on each region represents the number of the clusters. A cluster means a group of genes with more than 50% similarity and a difference of sequence length < 0.3.

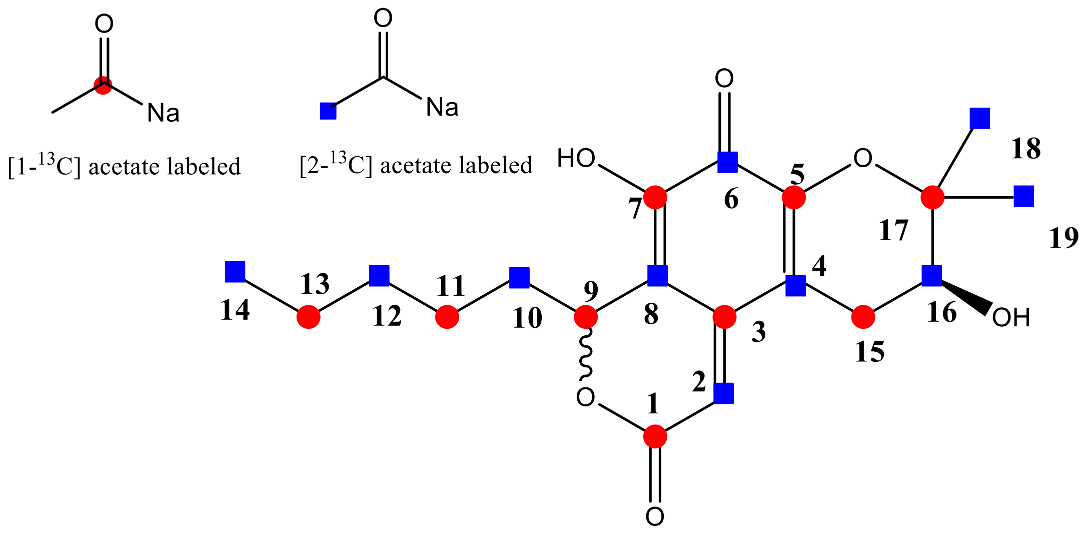

Supplementary Fig. 16. Results of isotopic incorporation for spiromarmycin biosynthesis. Sites of incorporation of ^{13}C labeled acetate into the spiromarmycin scaffold support the nature of its PKS-mediated assembly. Red spheres indicated carbons derived from the carbonyl moiety of acetate where as blue squares represent $^{13}CH_3$ derived carbons. Inspection of the ^{13}C NMR spectra of ^{13}C-labled spiromarmycin **1a** and **1b** revealed that: i) feeding of [1-^{13}C] acetate led to increases in the integration for resonances diagnostic for C_1, C_3, C_5, C_7, C_9, C_{11}, C_{13}, C_{15} and C_{17}; ii) feeding of [2-^{13}C]acetate led to increases in the integration for resonances diagnostic for C_2, C_4, C_6, C_8, C_{10}, C_{12}, C_{14}, C_{16}, C_{18}, and C_{19} (**Supplementary Figs. 26-29**) indicating that all 19 carbon atoms in **1a** and **1b** undergo labeling with either [1-^{13}C] acetate or [2-^{13}C] acetate. These data demonstrated, beyond any doubt, that the **1a** and **1b** are PKS-derived species.

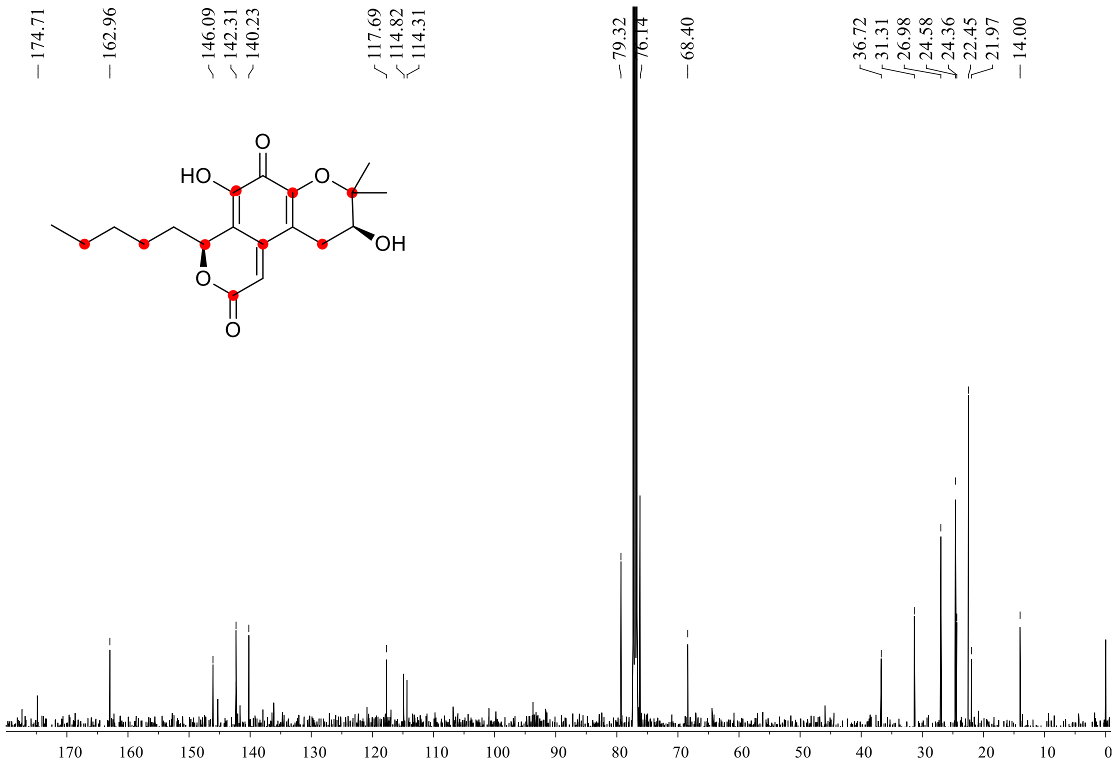

Supplementary Fig. 17. ^{13}C NMR spectrum (125 MHz) of (9*S*)-spiromarmycin **1a** labeled with [1-^{13}C]acetate in $CDCl_3$.

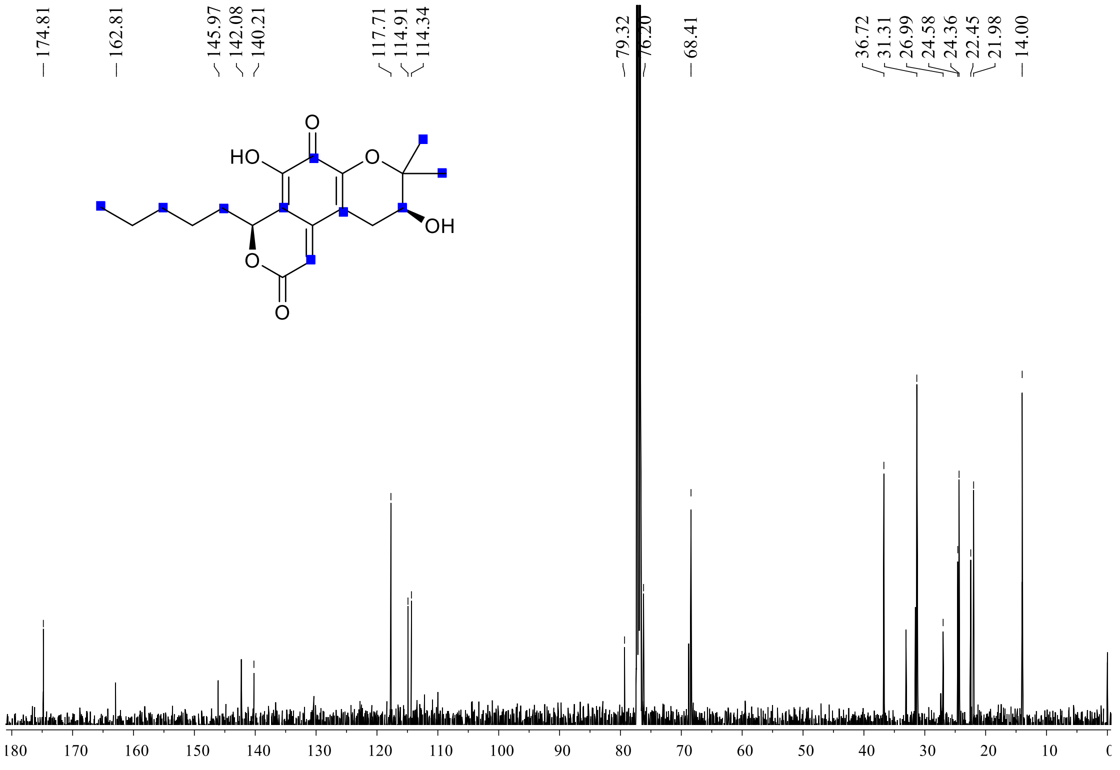

Supplementary Fig. 18. ^{13}C NMR spectrum (150 MHz) of (9*S*)-spiromarmycin **1a**.

labeled with [2-^{13}C]acetate in $CDCl_3$.

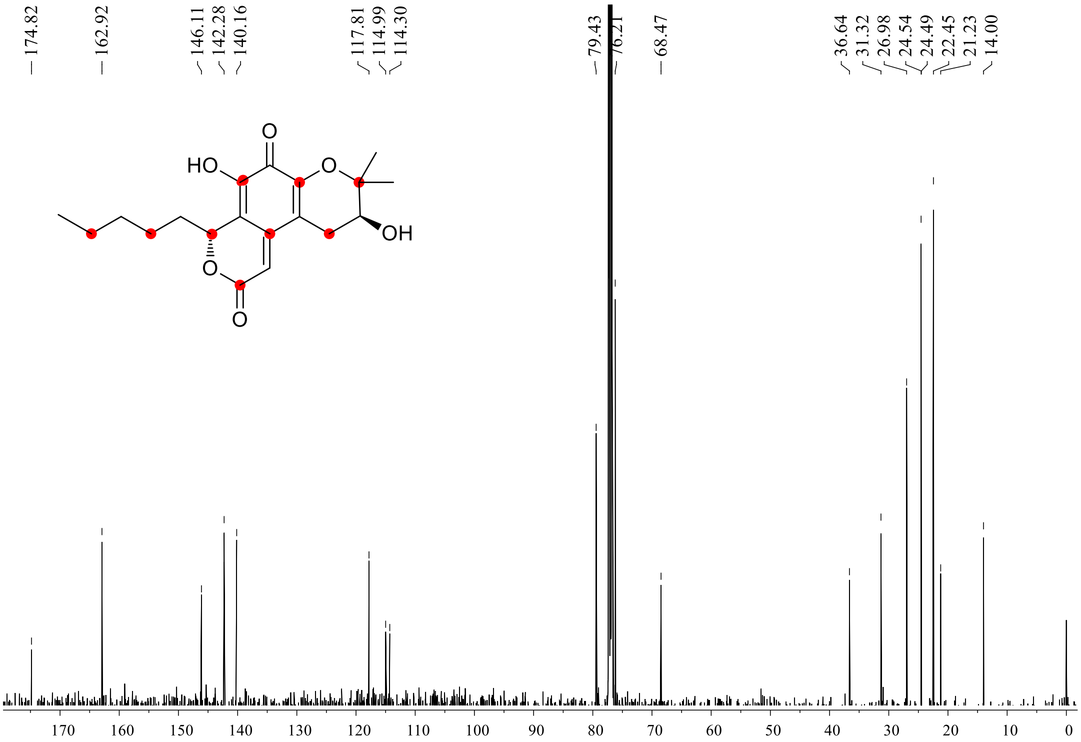

Supplementary Fig. 19. ^{13}C NMR spectrum (150 MHz) of (9*R*)-spiromarmycin **1b** labeled with [1-^{13}C]acetate in $CDCl_3$.

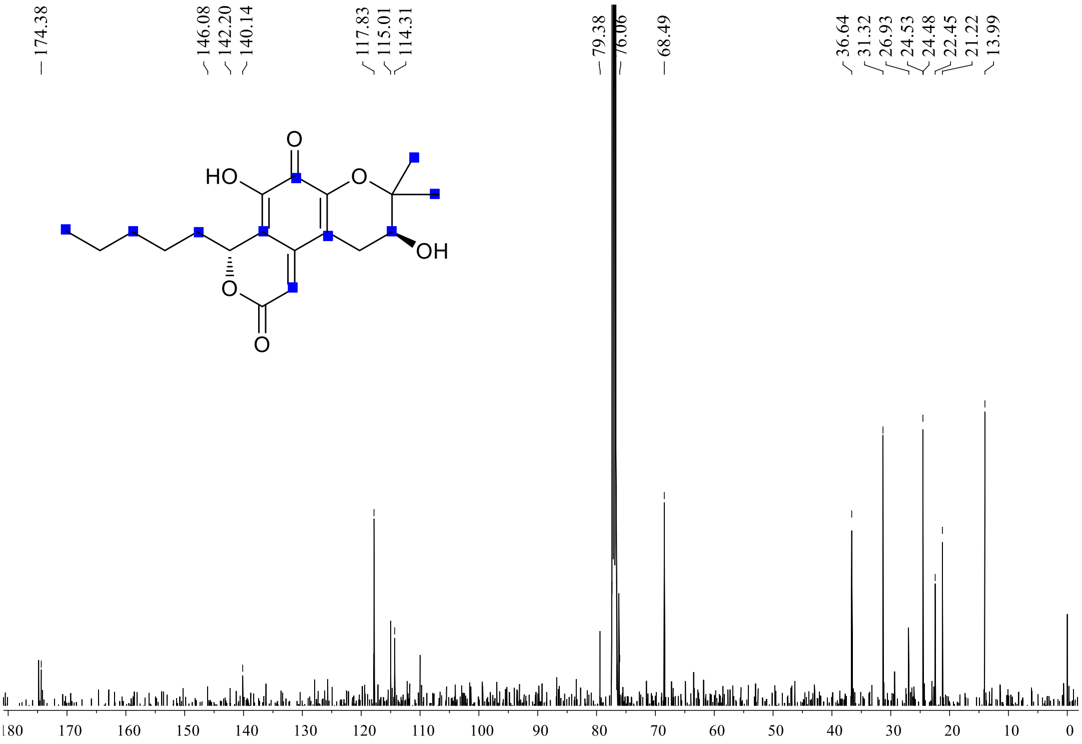

Supplementary Fig. 20. ^{13}C NMR spectrum (150 MHz) of (9*R*)-spiromarmycin **1b** [2-^{13}C]acetate in $CDCl_3$.

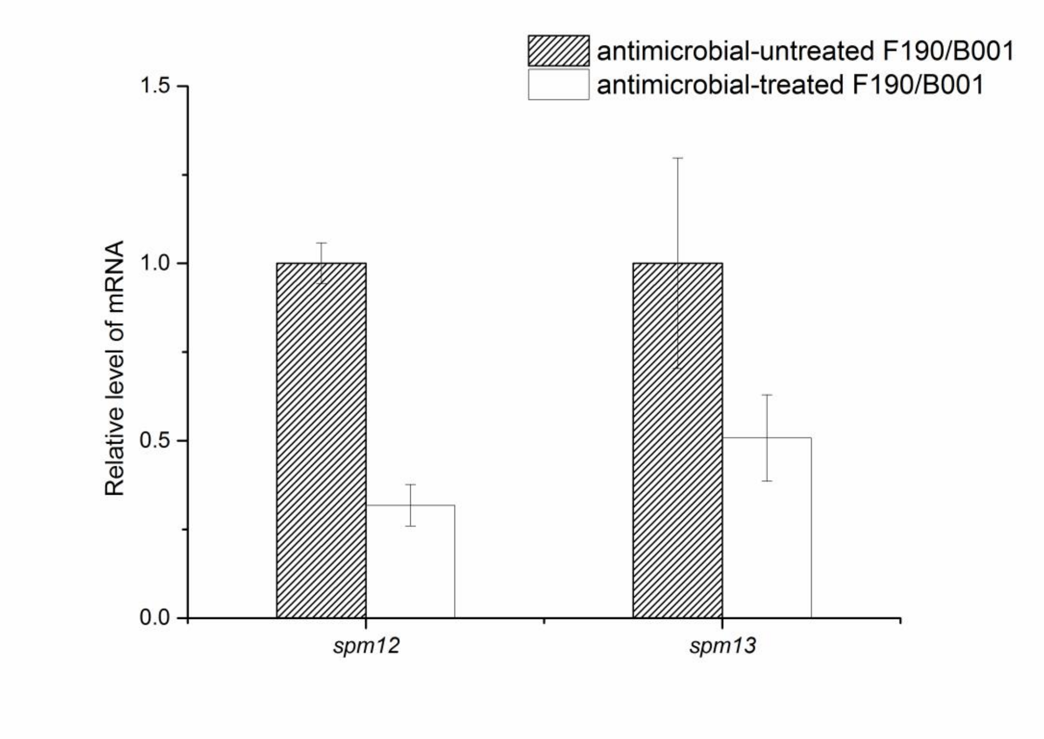

Supplementary Fig. 21. **Transcriptional analysis of two PKS genes, *spm12* and *spm13*, in the antimicrobial-untreated and antimicrobial-treated symbiont F190/B001 by quantitative real time RT-PCR.** The values were normalized to that of ITS1 and represented as means ± standard deviations (SD) (n = 3 biologically independent experiments); $p < 0.05$ (statistical analysis was performed in Microsoft Excel by Student's t-test).The amounts of each particular transcript in the antimicrobial-untreated F190/B001 were arbitrarily assigned as 1. The relative level of transcripts of *spm12* and *spm13* in the antimicrobial-treated symbiont F190/B001 is 32% and 50% to their counterparts in the antimicrobial-untreated symbiont F190/B001, respectively.

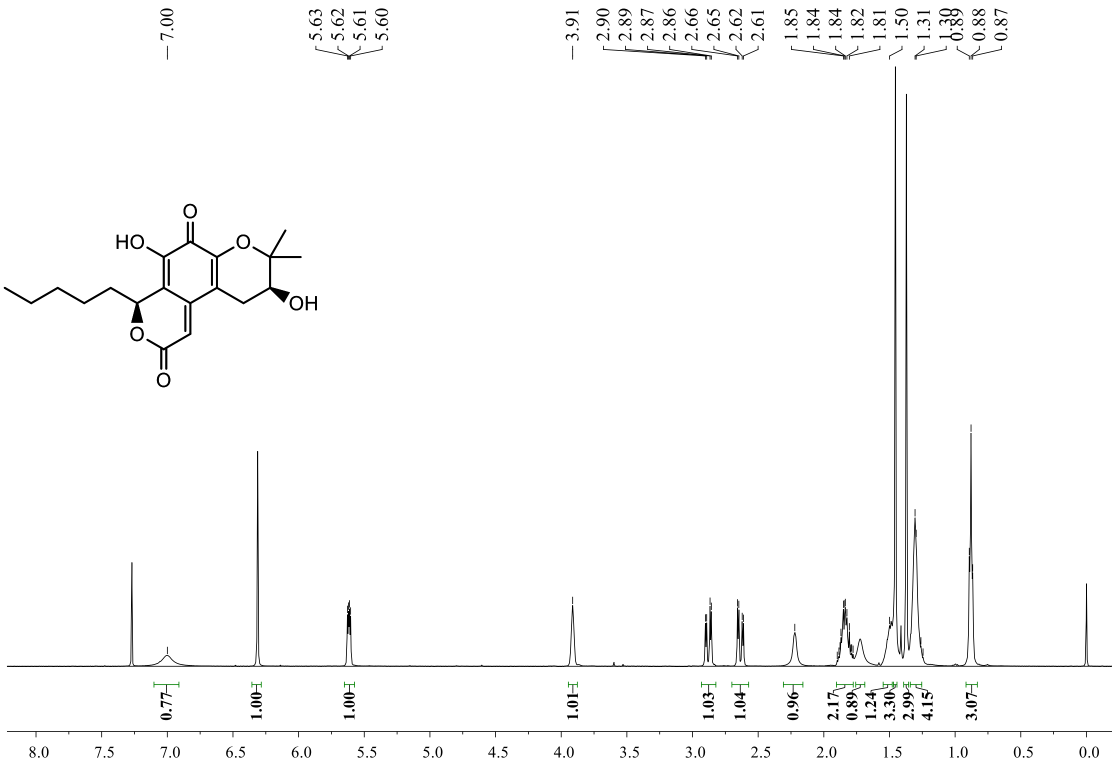

Supplementary Fig. 22. ^{1}H NMR spectrum (500 MHz) of (9*S*)-spiromarmycin **1a** in $CDCl_3$.

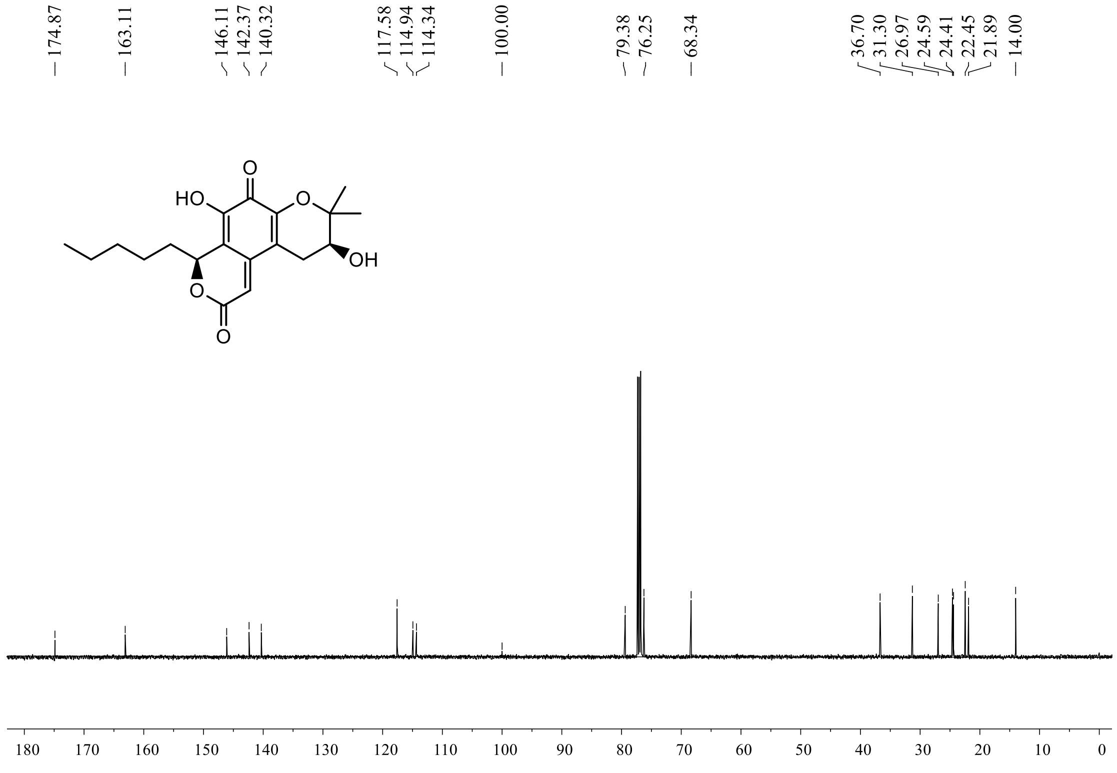

Supplementary Fig. 23. ^{13}C NMR spectrum (125 MHz) of (9*S*)-spiromarmycin **1a** in $CDCl_3$.

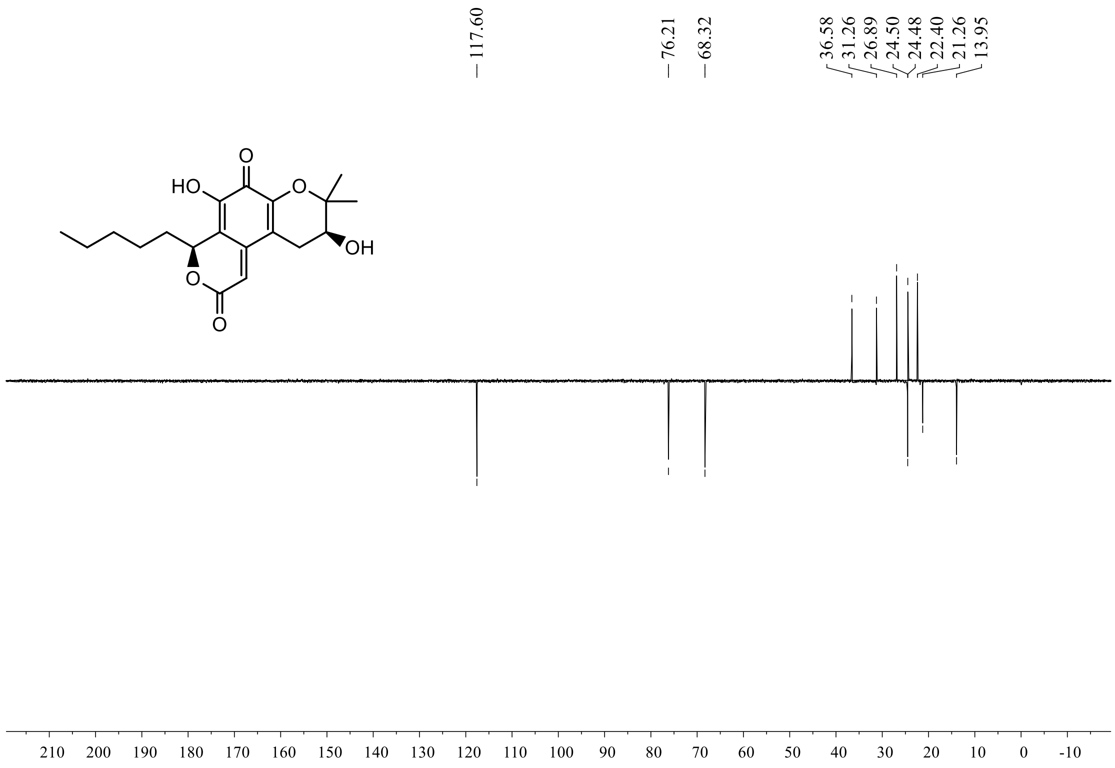

Supplementary Fig. 24. DEPT 135 spectrum (125 MHz) of (9*S*)-spiromarmycin **1a** in $CDCl_3$.

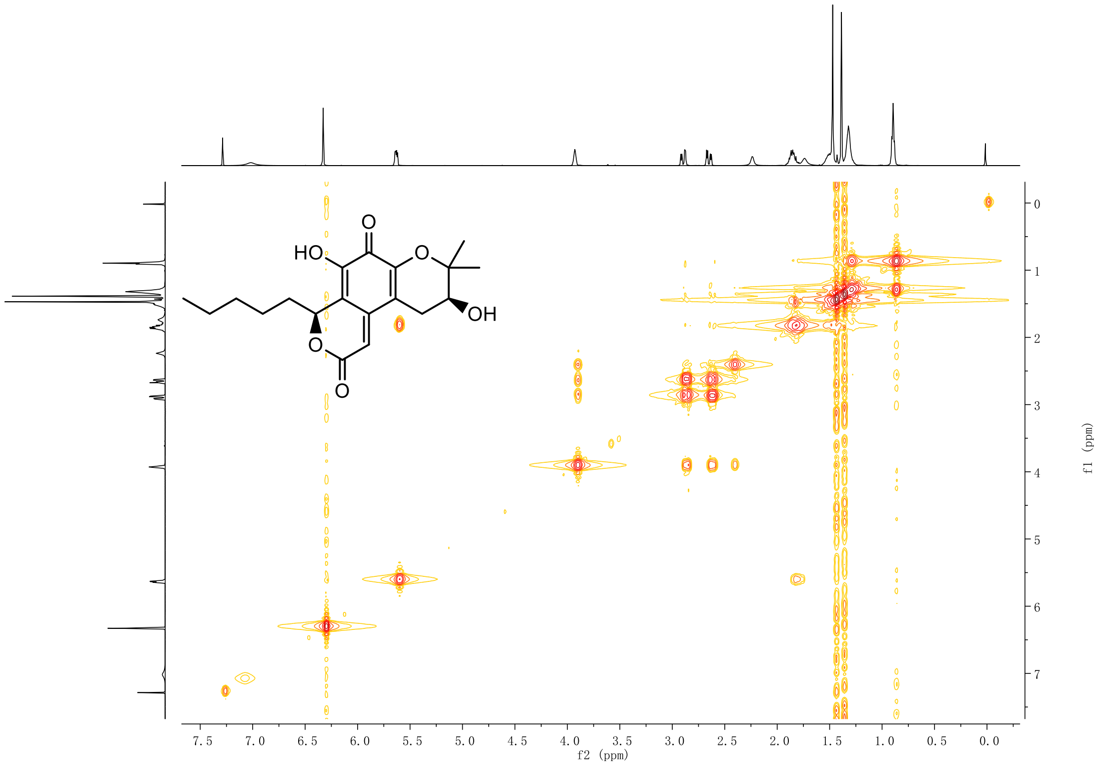

Supplementary Fig. 25. ^1H-^1H COSY spectrum of (9*S*)-spiromarmycin **1a** in $CDCl_3$.

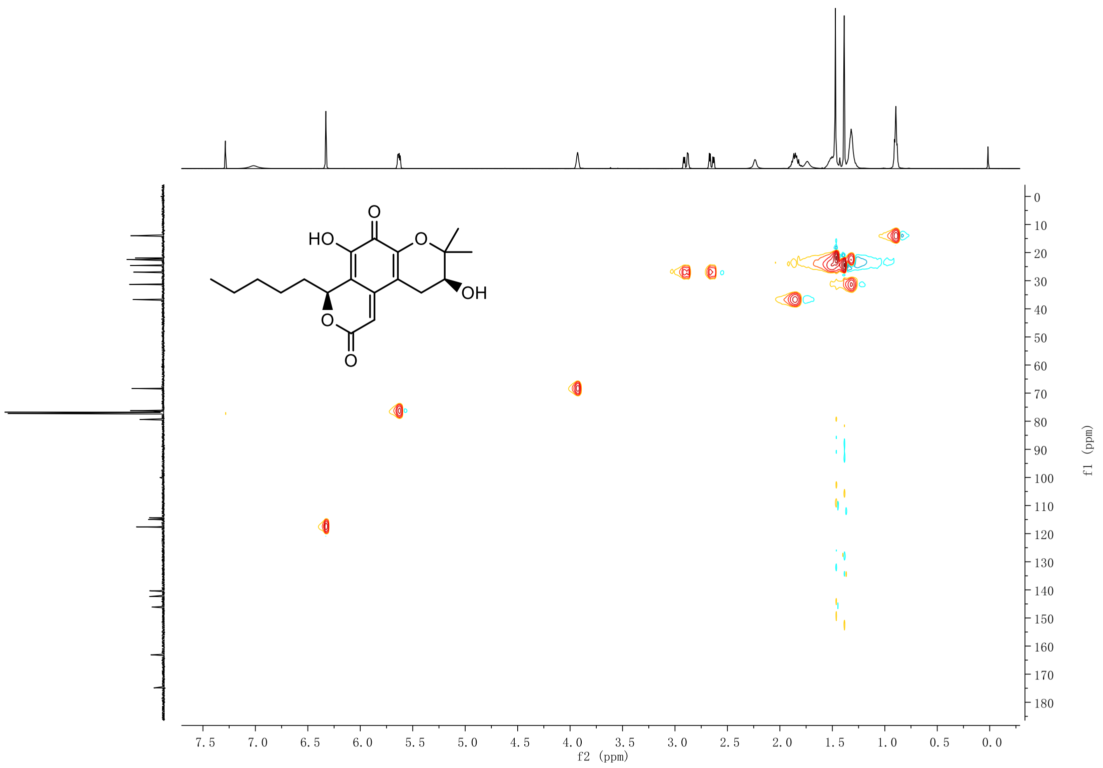

Supplementary Fig. 26. HMQC spectrum of (9*S*)-spiromarmycin **1a** in $CDCl_3$.

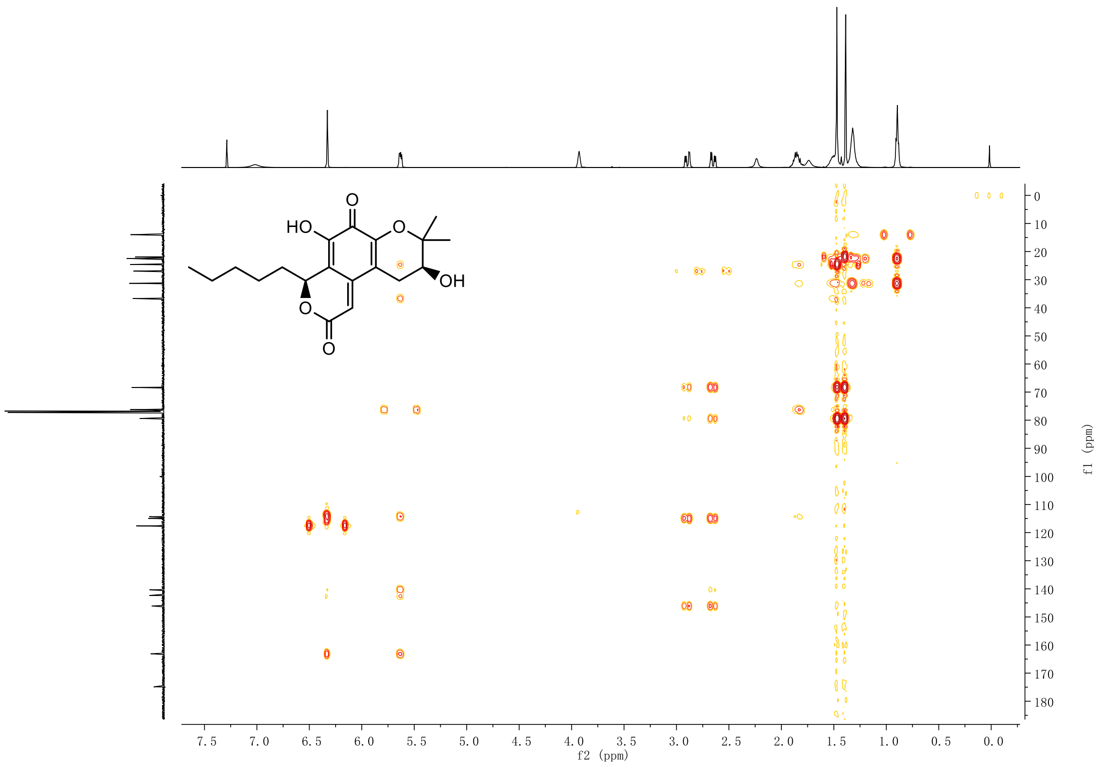

Supplementary Fig. 27. HMBC spectrum of (9*S*)-spiromarmycin **1a** in $CDCl_3$.

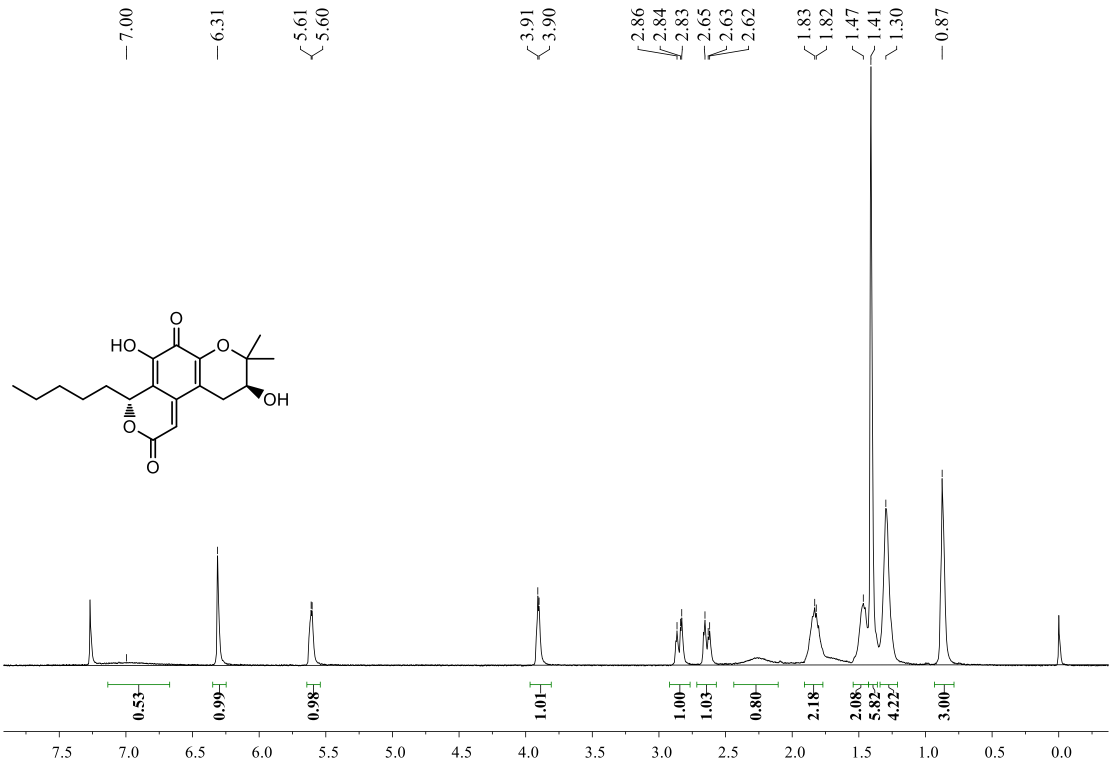

Supplementary Fig. 28. ^{1}H NMR spectrum of (9*R*)-spiromarmycin **1b** in $CDCl_3$.

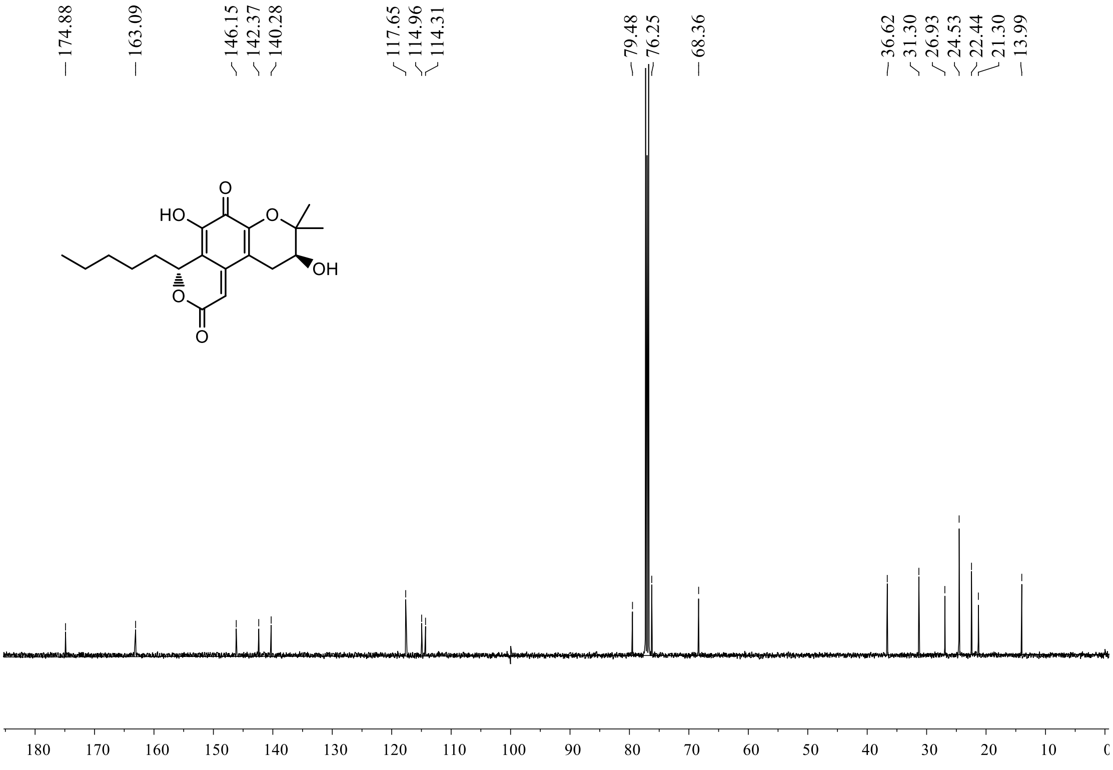

Supplementary Fig. 29. ^{13}C NMR spectrum (125 MHz) of (9*R*)-spiromarmycin **1b** in $CDCl_3$.

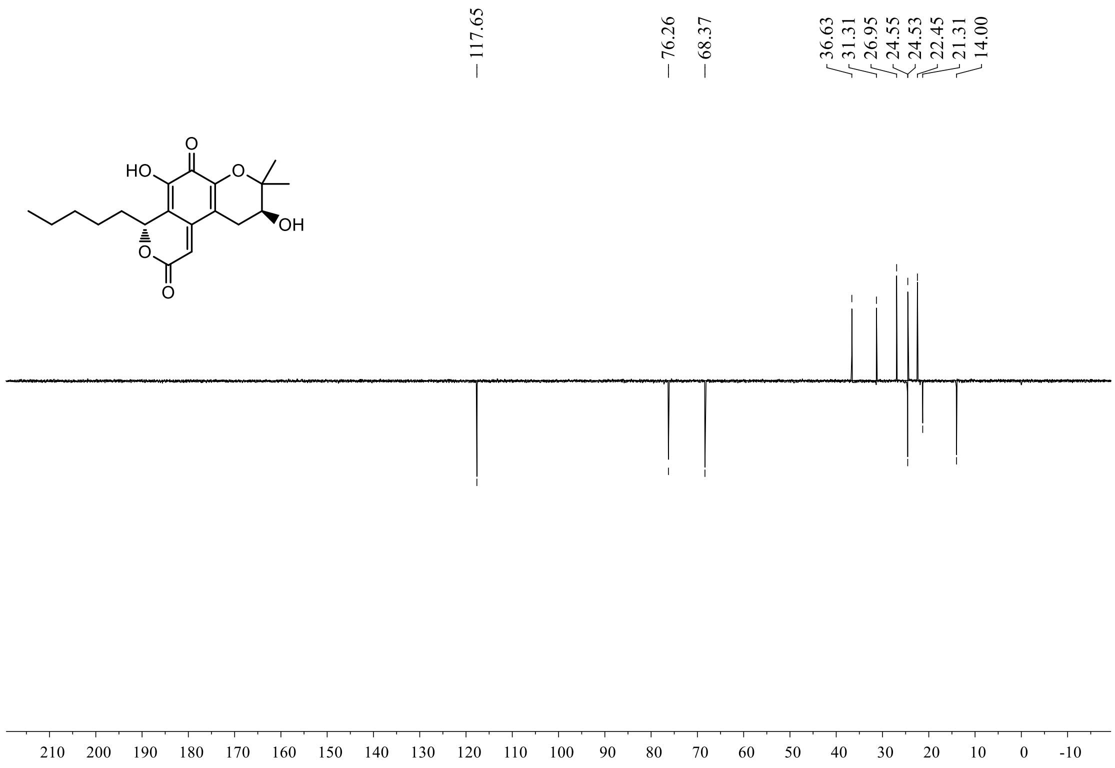

Supplementary Fig. 30. DEPT 135 spectrum (125 MHz) of (9*R*)-spiromarmycin **1b** in $CDCl_3$.

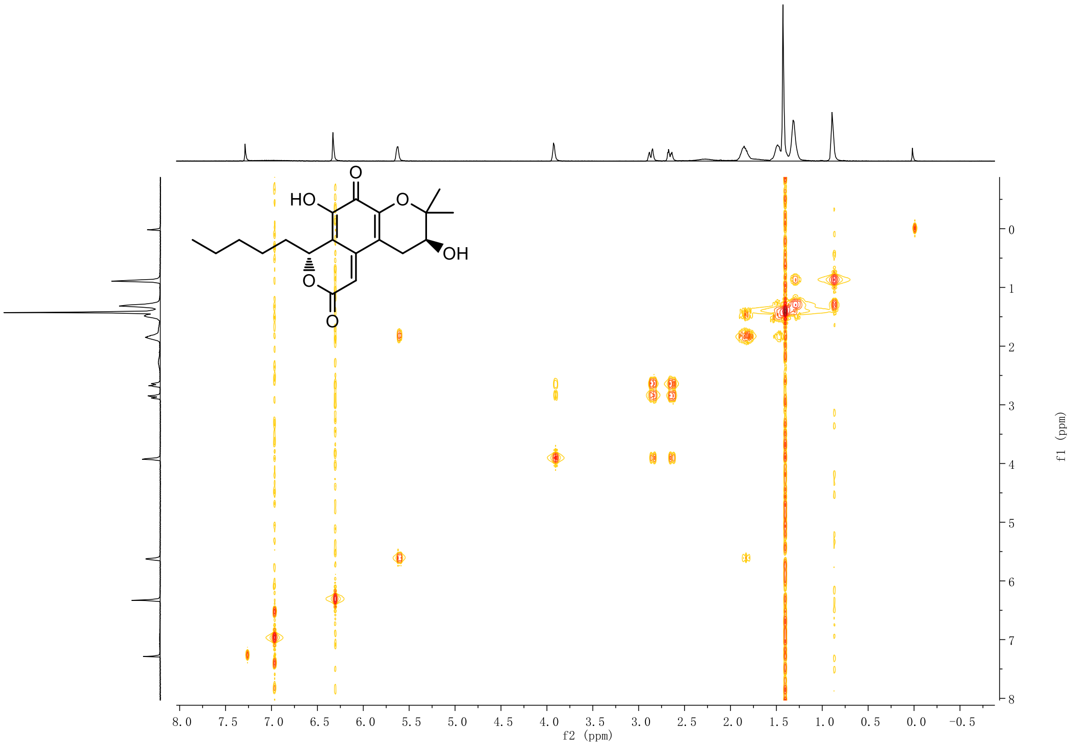

Supplementary Fig. 31. ^{1}H-^{1}H COSY spectrum of (9*R*)-spiromarmycin **1b** in $CDCl_3$.

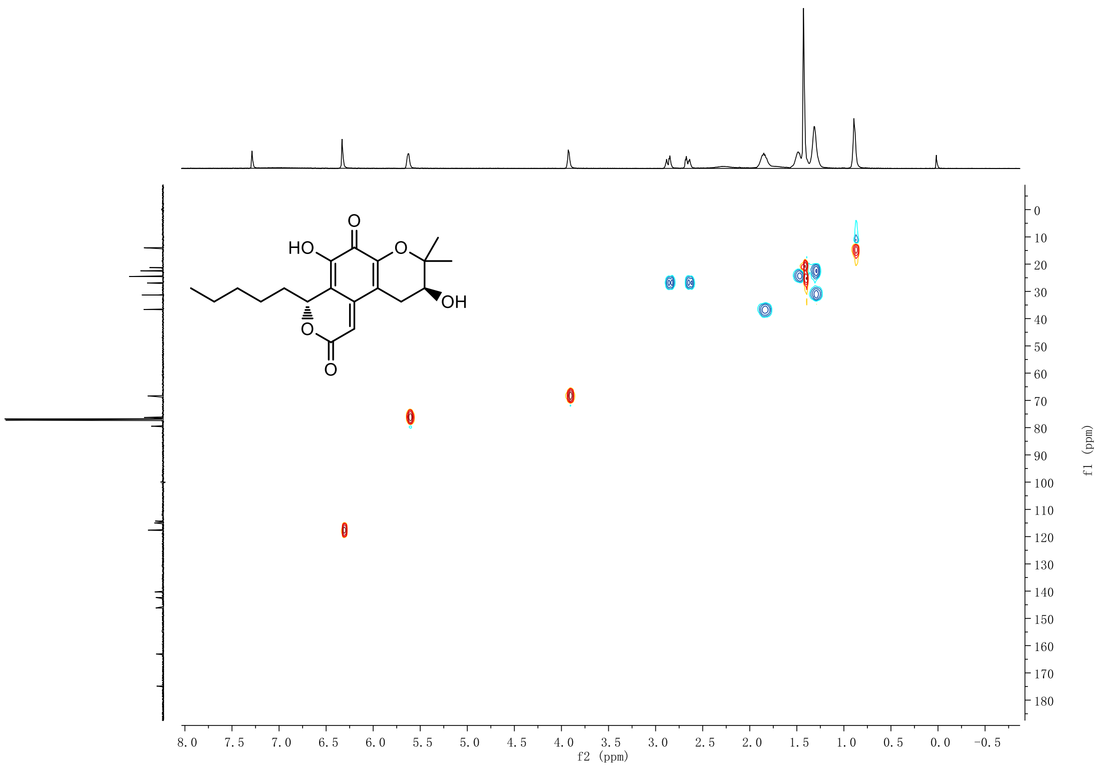

Supplementary Fig. 32. HMQC spectrum of (9*R*)-spiromarmycin **1b** in $CDCl_3$.

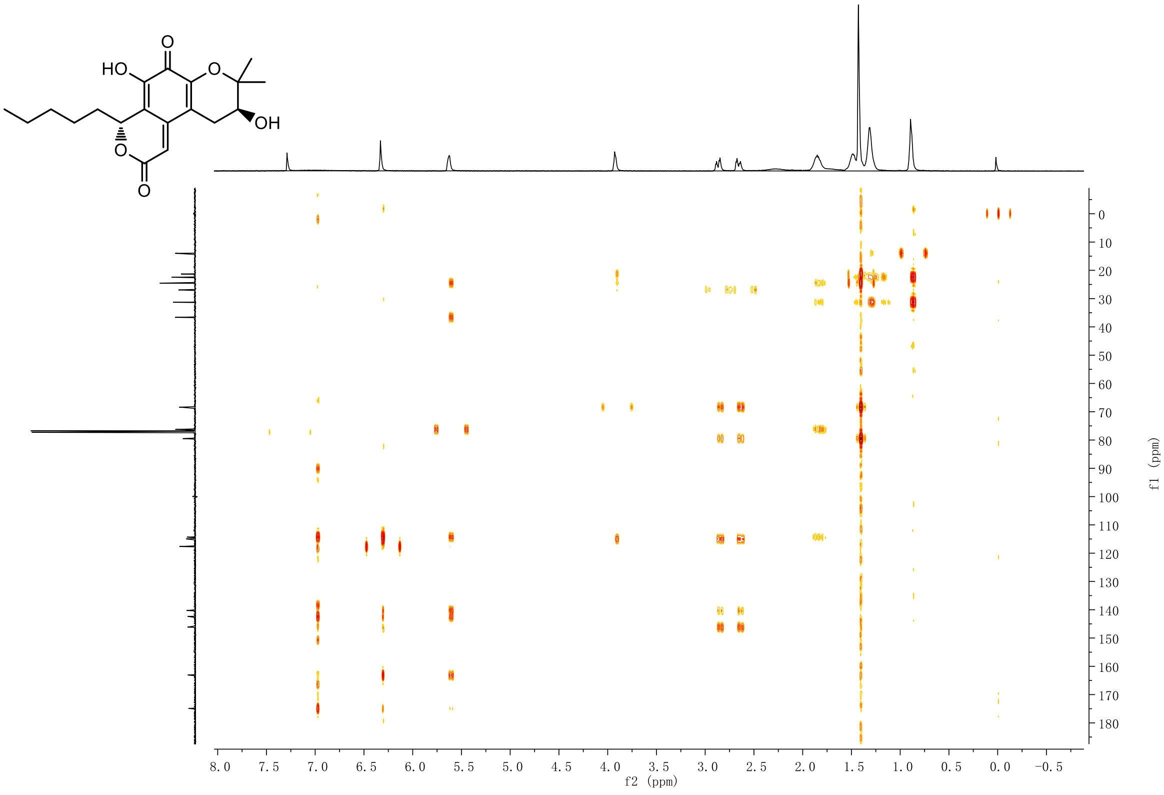

Supplementary Fig. 33. HMBC spectrum of (9*R*)-spiromarmycin **1b** in $CDCl_3$.

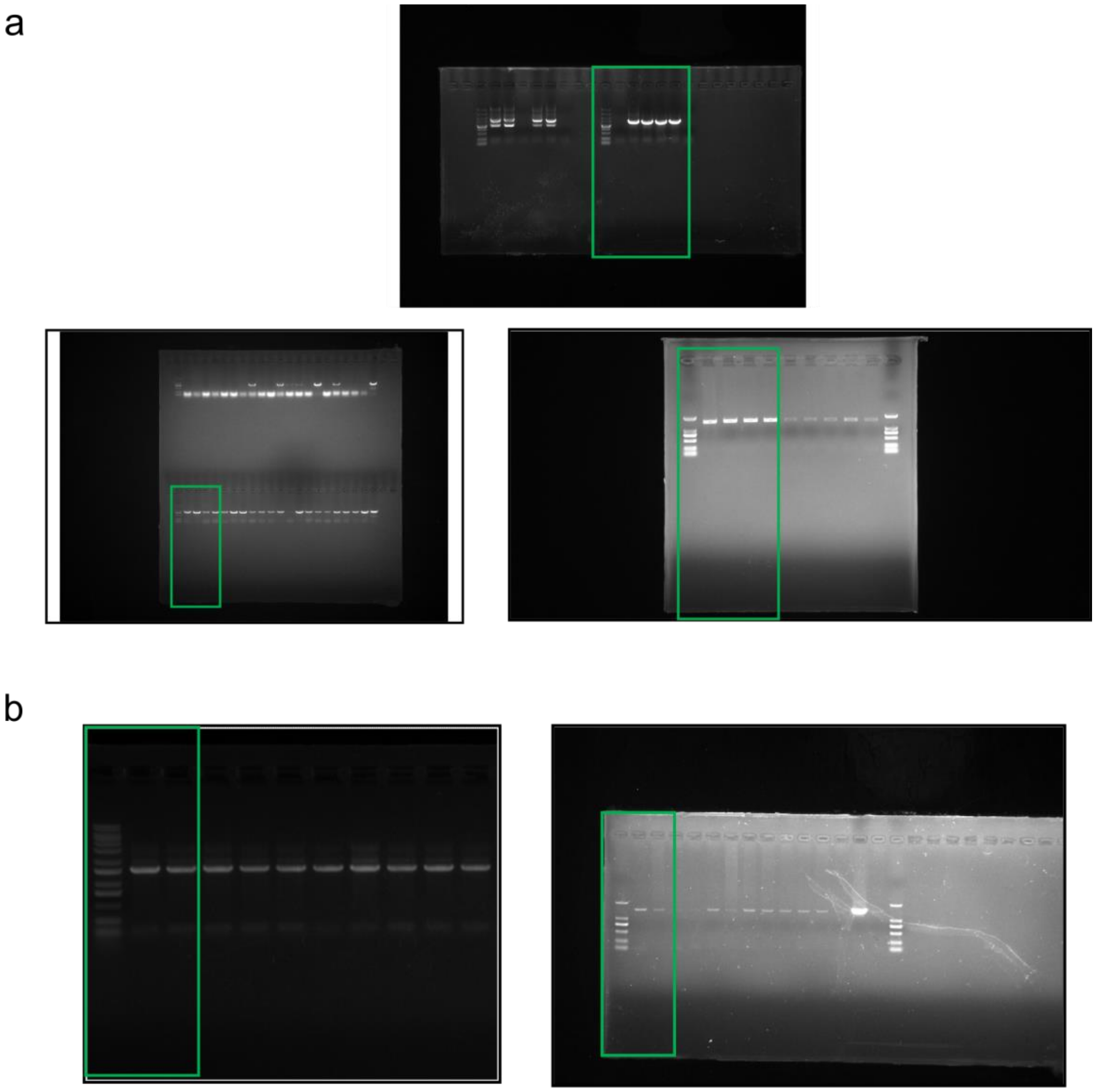

Supplementary Fig. 34. The uncut and full gels for the gels shown in the Supplementary Figs. 1 and 5. (a) The uncut and full gel of the gel shown in the Supplementary Fig. 1. (b) The uncut and full gel of the gel shown in the Supplementary Fig. 5.

Supplementary Tables

Supplementary Table 1. Summary of sensitivity of F190/B001 symbiont pairing and standalone bacterium B001 to different antibiotics. (in 50 μg/mL).

**Antibiotics**	**F190/B001**	**B001**
Antibacteria agents		
Ampicillin	R[a]	S[b]
Kanamycin	R	S
Trimethoprim	R	S
Chloromycetin	R	S
Ciprofloxacin hydrochloride	R	S
Antifungal agents		
Hygromycin	R	R
Bleomycin	R	R
Glyphosate	R	R
Nystatin	R	R
Geneticin	R	R

R[a] means: resistant to antibiotics; S[b] means: sensitive to antibiotics

Supplementary Table 2. The interconversion of **1a** to **1b** in 10 solvents as detected by HPLC (RT). See Supplementary Scheme 1 for proposed mechanism.

**Spiromarmycin 1a**	**Peak area ratio/%**		
	**8 h**	**14 h**	**24 h**
DMSO	49.46	49.63	49.20
Methanol	65.87	65.91	64.95
Methanol + acetone + water	49.41	49.23	48.58
Methanol + water	49.27	48.98	47.68
Methanol + water + acetic acid	84.76	81.61	77.82
Methanol + acetic acid	85.75	81.53	78.82
Water	49.85	49.56	49.14
Water + acetic acid	95.63	81.72	67.93
Acetonitrile + water	55.20	53.96	54.28
Acetonitrile + water + acetic acid	88.02	83.57	77.88

Supplementary Table 3. The interconversion of **1b** to **1a** in 10 solvents as detected by HPLC (RT). See Supplementary Scheme 1 for proposed mechanism. (peak area ratio/%).

**Spiromarmycin 1b**	**Peak area ratio/%**		
	8 h	14 h	24 h
DMSO	48.65	48.71	46.85
Methanol	36.27	32.28	24.44
Methanol + acetone + water	48.87	47.12	32.84
Methanol + water	49.06	47.77	34.84
Methanol + water + acetic acid	98.53	93.12	88.75
Methanol + acetic acid	95.27	93.50	83.02
Water	50.09	50.12	49.87
Water + acetic acid	94.42	87.45	71.16
Acetonitrile + water	50.09	45.28	45.50
Acetonitrile + water + acetic acid	99.39	98.07	88.45

Supplementary Table 4. Summary of ^{1}H, ^{13}C and HMBC-NMR data for **1a** and **1b** in $CDCl_3$

**Position**	**1a**			**1b**		
	δ_C	δ_H, **mult,** J **in Hz**	**HMBC**	δ_C	δ_H, **mult,** J **in Hz**	**HMBC**
1	163.1			163.1		
2	117.6	6.31, s	C-1,3	117.7	6.31, s	C-1,3
3	114.3			114.3		
4	146.1			146.2		
5	114.9			114.9		
6	174.9			174.9		
7	142.4			142.4		
8	140.3			140.3		
9	76.3	5.61, dd, 7.9, 4.2	C-11,10, 8,7,3,1	76.3	5.61, dd, 7.9, 4.2	C-11,10, 8,7,3,1
10	36.7	1.84, m	C-11,12,9	36.7	1.83, m	C-11,12,9
11	24.6	1.42, m	C-13	24.6	1.47, m	C-13
12	31.3	1.30, m	C-13	31.3	1.30, m	
13	22.5	1.30, m	C-12	21.3	1.30, m	C-12
14	14.0	0.88, t, 6.5	C-13,12	14.0	0.87, t, 6.5	C-13,12
15	27.0	2.63,dd,17.7,4.9; 2.88,dd,17.7,4.9	C-17,16, 5,4	27.0	2.63,m 2.84,m	C-17,16, 5,4
16	68.3	3.91, brs		68.4	3.90, brs	
17	79.4			79.5		
18	24.4	1.37, s	C-19, 17,16	26.9	1.41, s	C-19, 17,16
19	21.9	1.45, s	C-18,17,16	22.4	1.41, s	C-18,17,1 6
7-OH		7.0, m			6.88, m	C-7,6,3
16-OH		2.22, s			2.26, s	

Supplementary Table 5. Summary of antibacterial activities originally displayed by compound **1** during the course of initial bioactivity assessments.

**Pathogens**	**MIC (μg/mL)**					
	**1**	**1**	**Amp**[a]	**Kan**[b]	**Hyd**[c]	**Pol**[d]
Staphylococcus aureus ATCC 29213	+++	4.0	16.0	2.0	[e]NT	NT
MRSA	++	16.0	>128	>128	NT	NT
MRSA GDE4P037P	+++				NT	NT
MRSE	++	64.0	>128	>128	NT	NT
Staphylococcus aureus 29213	++	16.0	32.0	4.0	NT	NT
Staphylococcus aureus 1862	+	16.0	-	128.0	NT	NT
Staphylococcus aureus 669	++	8.0	-	1.0	NT	NT
Staphylococcus aureus 991	++	32.0	-	32.0	NT	NT
Staphylococcus aureus 16339	+++	32.0	-	2.0	NT	NT
Staphylococcus aureus 6917	+++	16.0	-	-	NT	NT
Staphylococcus aureus 16162	+++	16.0	-	-	NT	NT
Staphylococcus aureus 718306	+++	64.0	-	2.0	NT	NT
Staphylococcus aureus 745524	+++	16.0	-	-	NT	NT
Staphylococcus aureus MRSA GDE4P037P	+++	64.0	-	-	NT	NT
Staphylococcus aureus (cfr) GDQ6P012P	++	16.0	-	4.0	NT	NT
Staphylococcus cohnii DKG4	+	64.0	-	-	NT	NT
Staphylococcus simulans AKA1	++	-	-	-	NT	NT
Vibrio alginolyticus XSBZ14	+++	32.0	-	4.0	NT	NT
Enterococcus faecalis ATCC 29212	++	16.0	-	-	NT	NT
Enterococcus faecalis 5F137C	++	64.0	-	NT	NT	NT
Enterococcus gallinarum 5F52C	++	32.0	8.0	8.0	NT	NT
Enterococcus faecium 35682	+++	32.0	2.0	NT	NT	NT
Enterococcus faecium 36235	+++	16.0	-	NT	NT	NT
Enterococcus faecium 36711	+++	32.0	1.0	NT	NT	NT
Enterococcus faecium 36950	+++	32.0	0.5	NT	NT	NT
Clostridium perfringens FSKP20	+	-	-	-	NT	NT
Erwinia carotovora	++		-	2.0	NT	NT
Bacillus subtilis	-			16.0	NT	NT
Micrococcus luteus	++	8.0	2.0	0.5	NT	NT
Bacillus thuringiensis	++	16.0	-	64.0	NT	NT
Escherichia coli ATCC 25922	-		NT	NT	NT	NT
Escherichia coli Eco 920	-		NT	NT	NT	NT
Escherichia coli	-		NT	NT	NT	NT
Escherichia coli 761	-		NT	NT	NT	NT
Escherichia coli						
Escherichia coli 16369	+++	-	NT	NT	32.0	4.0
Escherichia coli 16447	+	-	NT	NT	0.25	4.0

Escherichia coli 737720	+	-	NT	NT	8.0	4.0
Escherichia coli 16351	+	-	NT	NT	64.0	4.0
Escherichia coli ATCC 13124	+		NT	NT	32.0	-
Escherichia coli (E11)	+		NT	NT	8.0	2.0
Escherichia coli SHPP45	-		NT	NT	NT	NT
Escherichia coli (ST117) SC0517	-		NT	NT	NT	NT
Acinetobacter baumannii ATCC 19606	+	128.0	NT	NT	8.0	4.0
Acinetobacter baumannii ADR-2	-		NT	NT	NT	NT
Acinetobacter baumannii Abs-8	-		NT	NT	NT	NT
Acinetobacter baumannii 15122	+	-	NT	NT	32.0	4.0
Acinetobacter baumannii 15407	+	-	NT	NT	32.0	4.0
Acinetobacter baumannii 15199	+	-	NT	NT	32.0	4.0
Acinetobacter baumannii 14892	+	-	NT	NT	16.0	4.0
Acinetobacter baumannii 15532	-		NT	NT		
MRPA/*Pseudomonas aeruginosa*	++	-	NT	NT	4.0	4.0
*Pseudomonas aeruginosa*14472	+	-	NT	NT	2.0	4.0
*Pseudomonas aeruginosa*6709	-		NT	NT	NT	NT
*Pseudomonas aeruginosa*13827	+	-	NT	NT	1.0	2.0
*Pseudomonas aeruginosa*9747	+	-	NT	NT	1.0	2.0
*Pseudomonas aeruginosa*15690	+	-	NT	NT	1.0	4.0
Klebsiella pneumoniae ATCC 13883	+	-	NT	NT	0.25	4.0
Klebsiella pneumoniae 15580	++	-	NT	NT	4.0	2.0
Salmonella typhimurium SH138	-		NT	NT	NT	NT
Alcaligenes faecali SCSIO B001	-		NT	NT	NT	NT

[a]Ampicilin, [b]kanamycin, [c]hydrochloride and [d]polymyxin were co-assayed as a positive control. [e]NT No tested. -: no activity (zone of inhibition <10 mm); +: activity (zone of inhibition: 12~15 mm); ++: good activity (zone of inhibition: 15~20 mm); +++: very good activity (zone of inhibition>20 mm).

The bioactivity data showed that spiromarmycin exhibited potent antibacterial activities against *S. aureus* ATCC 29213 with MIC = 4.0 µg/mL and MRSA with MIC = 16 µg/mL. The spiromarmycin also exhibited potent antibacterial activities against *Vibrio alginolyticus* XSBZ14 with MIC = 32.0 µg/mL, *E. faecali* ATCC 29212 with MIC = 16 µg/mL, one clinical *M. luteus* with MIC = 8.0 µg/mL, and one clinical *B. thuringiensis* with MIC = 16.0 µg/mL, respectively. Weak activities against gram-negative *Escherichia coli* (MIC = 128~512 µg/mL) also were detected during activity screening for spiromarmycin. Spiromarmycin showed good activities against *Klebsiella pneumoniae* ATCC 13883 with MIC = 128 µg/mL, weak antibacterial activities against *Acinetobacter baumannii* (ATCC 19606) as well as *Pseudomonas aeruginosa* and another four clinical isolates with MIC = 128~512 µg/mL. Spiromarmycin was found to have no antibacterial activities against the poultry pathogen *Salmonella typhimurium* SH138, *Proteus mirabilis* SG0508, *Salmonella heidelberg* SH36. These data indicate that spiromarmycin displays good broad-spectrum antibacterial activities and likely enables symbiont F190/B001 to drive away other prokaryotic microorganisms from the symbiont’s surroundings.

Supplementary Table 6. Zone of inhibition and MIC of spiromarmycin against the *Candida albicans.*

**Pathogens**		**MIC(μg/mL)**		
	**Spiromarmycin**	**Spiromarmycin**	**Amphotericin B**[a]	**Fluconazole**[a]
Candida albicans ATCC 96901	+	16.0	0.5	64.0
Candida albicans 173202399	+	64.0	0.5	128.0
Candida albicans 173202375	+	2.0	0.25	0.25
Candida albicans 174105146	+	1.0	0.25	0.25
Candida albicans 173202416	+	2.0	0.25	0.25
Candida albicans 173202351	+	16.0	0.5	0.5
Candida albicans 98001	+	128.0	64	0.25
Candida albicans	+++	64.0	-	-

[a]Amphotericin B and Fluconazole were co-assayed as a positive control. [b]NT No tested. -: no activity (zone of inhibition<10 mm); +: activity (zone of inhibition: 12~15 mm); ++: good activity (zone of inhibition: 15~20 mm); +++: very good activity (zone of inhibition >20 mm).

The results of activity test showed that spiromarmycin exhibited potent antifungal activities against fluconazole-resistant *C. albicans* ATCC 96901 (MIC = 16 μg/mL) and three clinical *C. albicans* (173202375: MIC = 2 μg/mL; 174105146: MIC = 1.0 μg/mL; 173202416: MIC = 2 μg/mL), and showed weak antifungal activities against a number of other isolates.

Supplementary Table 7. Summary of *A. faecali* SCSIO B001 genome features.

**Features**	**Value**
Genome size (bp)	4029778
G + C content (%)	56.77
Gene num	3680
Gene total length	3559956
Gene average length	967.
Gene density (Kb)	0.913
GC content in gene region (%)	57.7
Gene/Geonme (%)	88.3
Intergenetic region length	469822
GC content in intergenetic region (%)	49.7
Intergenetic length/Genome (%)	11.7
rRNAs	9
tRNAs	56
Secondary metabolites clusters	6

Supplementary Table 8. Summary of *Spiromastix* sp. SCSIO F190 genome features.

Features	Value
Total num (#)	32
Total Length(bp)	38,211,887
Average Length(bp)	1,194,121
N50 length（bp）	5,195,063
N90 length（bp）	2,393,209
Max length（bp）	12,632,200
Min length（bp）	3,893
GC Content(%)	44.76
N rate (%)	0
Gene stat/Total length	13,521,685
Gene stat/Total number	8,334
Gene stat/Average length	1,622.7
Exons stat/Total length	11,976,897
Exons stat/Total number	26,364
Exons stat/Average length	454.9
CDS stat/Total length	11,976,897
CDS stat/Total number	8,334
CDS stat/Average length	1,437.1
Intron stat/Total length	1,544,788
Intron stat/Total number	18,030
Intron stat/Average length	85.68
Gene/Geonme(%)	68.66
tRNA	44
5S rRNA	12
5.8S rRNA	0
18S rRNA	2
28S rRNA	1
sRNA	2
sn RNA	12
miRNA	0
Secondary metabolites clusters	23

Supplementary Table 9. Genetic statistics of Core-pan gene of *Spiromastix* sp. SCSIO F190 through the comparison of the protein sequences with selected ten strains.

All gene number (#)	Core gene number (#)	Pan gene number (#)
106,324	51,591	761

Supplementary Table 10. Functional assignments for genes identified in Type I-PKS cluster identified in the *A. faecali* SCSIO B001 genome.

ORF	Size[a]	Proposed function	Protein homologue	Identity/ Similarity (%)
orf1	317	Polysaccharide deacetylase	WP_051010473.1	88/100
orf2	486	Mannose-1-phosphate guanylyltransferase/mannose-6-phosphate isomerase	WP_074904564.1	68/98
orf3	331	Hypothetical protein	WP_046771495.1	49/96
orf4	44	Unknown	-	-
orf5	386	Glycosyl transferases group 1	SEG20075.1	50/99
orf6	1191	Glycosyltransferase involved in cell wall bisynthesis	SEG20091.1	42/74
orf7	1119	Methyltransferase type 12	WP_059318160.1	63/99
orf8	421	UDP-N-acetyl-D-mannosamine dehydrogenase	WP_042485831.1	99/100
orf9	373	UDP-N-acetylglucosamine 2-epimerase (non-hydrolyzing)	WP_045929494.1	99/100
orf10	272	Hypothetical protein	WP_083054875.1	46/91
orf11	396	Chain-length determining protein	KGP02092.1	91/100
orf12	220	ABC transporter ATP-binding protein	WP_083054877.1	97/100
orf13	262	ABC transporter	WP_035270125.1	91/100
orf14	385	Capsular biosynthesis protein	ALO38980.1	100/100
orf15	446	8-amino-7-oxononanoate synthase	WP_045929490.1	99/100
orf16	2524	Type I polyketide synthase (KS, AT, DH, ER, KR, ACP)	OSZ34143.1	99/100
orf17	496	Permease	WP_063691891.1	99/100
orf18	287	Eama/rhat family transporter	WP_042485819.1	100/100
orf19	463	Gntr family transcriptional regulator	WP_086061202.1	100/100
orf20	301	Eama family transporter	WP_080723773.1	100/100
orf21	519	Hypothetical protein	WP_063691897.1	95/100
orf22	247	DUF2094 domain-containing protein	WP_086061200.1	98/100
orf23	1199	Hypothetical protein	WP_063691903.1	99/100
orf24	418	Hypothetical protein	ARP55284.1	99/100
orf25	440	Type VI secretion protein	WP_042485801.1	100/100
orf26	167	Type VI secretion lipoprotein	WP_042485798.1	99/100
orf27	129	Hypothetical protein	WP_086069083.1	100/100
orf28	196	Hypothetical protein	WP_063691916.1	99/100
orf29	347	Hypothetical protein	WP_086069085.1	99/100
orf30	628	Hypothetical protein	WP_042485788.1	99/100
orf31	180	Hypothetical protein	WP_045929477.1	100/100
orf32	160	Hcp1 family type VI secretion system effector	WP_035270090.1	99/100

[a]Size in units of amino acids (aa)

Supplementary Table 11. Functional assignments for genes composing the spiromarmycin (*spm*) biosynthetic gene cluster.

ORF	Size[a]	Proposed function	Protein homologue	Identity/ Similarity (%)
spm1	561	GMC oxidoreductase	OTA96323.1	53/98
spm2	261	Hypothetical protein	OJJ36822.1	36/86
spm3	229	Uridylate kinase	XP_002621750.1	71/96
spm4	644	Eukaryotic translation initiation factor 3 subunit M	XP_001240338.1	68/73
spm5	95	Thioesterase	EGE87111.1	44/66
spm6	973	Hypothetical protein	EYE92270.1	42/86
spm7	471	Dimethylallyl tryptophan synthase glid1	XP_018028965.1	46/83
spm8	244	FAD binding domain protein	XP_007817537.1	37/95
spm9	173	Hypothetical protein	OBT88109.1	26/76
spm10	626	multicopper oxidase	XP_026623687.1	44/93
spm11	299	Oxidoreductase cipa-like, putative	XP_007809030.1	51/96
spm12	1798	Non-reducing iterative polyketide synthase	KKY30996.1	75/100
spm13	2362	Reducing iterative polyketide synthase	KKY30995.1	79/100
spm14	336	Salicylate hydroxylase	AMR44277.1	77/98
spm15	592	MFS multidrug transporter	AMR44276.1	69/100
spm16	294	Short chain dehydrogenase reductase	AMR44278.1	77/92
spm17	111	Cupin domain-containing protein	OCW32922.1	55/93
spm18	129	Zinc finger protein	AMR44275.1	54/82
spm19	363	Tyrosyl-trna synthetase	KKK21726.1	69/88
Spm20	327	Thioesterase	XP_002583008.1	65/96
spm21	978	Trehalose 6-phosphate synthase	KLJ09831.1	63/99

[a]Size in units of amino acids (aa)
