# Supplementary material for: Upregulation of a marine fungal biosynthetic gene cluster by an endobacterial symbiont
Source: Commun Biol. 2020 Sep 23;3:527. doi: 10.1038/s42003-020-01239-y (PMC7511336; doi:10.1038/s42003-020-01239-y)
Supplement: Supplementary file 2 — Description of Additional Supplementary Files [file 42003_2020_1239_MOESM2_ESM.pdf]

Description of Additional Supplementary Files

File Name: Supplementary Data 1
Description: HPLC analyses of the PDA fermentation butanone extracts (Fig. 2c). Trace i: the source data for the HPLC plot of the negative control of the symbiont F190/B001; trace ii: the source data for the HPLC plot of the symbiont F190/B001 treated with ampicillin; trace iii: the source data for the HPLC plot of the symbiont F190/B001 treated with kanamycin; trace iv: the source data for the HPLC plot of the symbiont F190/B001 treated with chloromycetin; trace v: the source data for the HPLC plot of the symbiont F190/B001 treated with ciprofloxacin; trace vi: the source data for the HPLC plot of the symbiont F190/B001 after treated with four kinds of antibiotics and retrained on a ISP2 plate containing ciprofloxacin hydrochloride consecutively; trace vii: the source data for the HPLC plot of the symbiont F190/B001 precipitate out the irregular brown plaques; trace viii: the source data for the HPLC plot of the purified compounds spiromarmycin; trace ix: the source data for the HPLC plot of *A. faecalis* SCSIO B001.
