# Supplementary material for: Upregulation of a marine fungal biosynthetic gene cluster by an endobacterial symbiont
Source: Commun Biol. 2020 Sep 23;3:527. doi: 10.1038/s42003-020-01239-y (PMC7511336; doi:10.1038/s42003-020-01239-y)
Supplement: Supplementary file 4 — Reporting summary [file 42003_2020_1239_MOESM4_ESM.pdf]

nature research

Corresponding author(s): Jianhua Ju

Last updated by author(s): Aug 8, 2020

Reporting Summary

Nature Research wishes to improve the reproducibility of the work that we publish. This form provides structure for consistency and transparency in reporting. For further information on Nature Research policies, see our Editorial Policies and the Editorial Policy Checklist.

Statistics

For all statistical analyses, confirm that the following items are present in the figure legend, table legend, main text, or Methods section.

n/a	Confirmed	
☐	☒	The exact sample size (*n*) for each experimental group/condition, given as a discrete number and unit of measurement
☐	☒	A statement on whether measurements were taken from distinct samples or whether the same sample was measured repeatedly
☒	☐	The statistical test(s) used AND whether they are one- or two-sided<br>*Only common tests should be described solely by name; describe more complex techniques in the Methods section.*
☒	☐	A description of all covariates tested
☒	☐	A description of any assumptions or corrections, such as tests of normality and adjustment for multiple comparisons
☒	☐	A full description of the statistical parameters including central tendency (e.g. means) or other basic estimates (e.g. regression coefficient) AND variation (e.g. standard deviation) or associated estimates of uncertainty (e.g. confidence intervals)
☒	☐	For null hypothesis testing, the test statistic (e.g. *F*, *t*, *r*) with confidence intervals, effect sizes, degrees of freedom and *P* value noted<br>*Give P values as exact values whenever suitable.*
☒	☐	For Bayesian analysis, information on the choice of priors and Markov chain Monte Carlo settings
☒	☐	For hierarchical and complex designs, identification of the appropriate level for tests and full reporting of outcomes
☒	☐	Estimates of effect sizes (e.g. Cohen's *d*, Pearson's *r*), indicating how they were calculated

Our web collection on statistics for biologists contains articles on many of the points above.

Software and code

Policy information about availability of computer code

Data collection	We did not use such software.
Data analysis	The following softwares were used to analyze the genome sequencing raw data: SOAP de novo v2.04, Celera Assembler 8.0 and SOPA Gap Closer v1.12.

For manuscripts utilizing custom algorithms or software that are central to the research but not yet described in published literature, software must be made available to editors and reviewers. We strongly encourage code deposition in a community repository (e.g. GitHub). See the Nature Research guidelines for submitting code & software for further information.

Data

Policy information about availability of data

All manuscripts must include a data availability statement. This statement should provide the following information, where applicable:

- Accession codes, unique identifiers, or web links for publicly available datasets
- A list of figures that have associated raw data
- A description of any restrictions on data availability

The genomes of A. faecalis SCSIO B001 and Spiromastix sp. F190 have been submitted to the NCBI and are covered by the NCBI BioProject numbers PRJNA598809 and PRJNA598808, respectively. Crystallographic data for spiromarmycin have been deposited with the Cambridge Crystallographic Data Center with the deposition number CCDC 2016950. Source data underlying the plots shown in Fig. 2c has been provided in Supplementary Data 1. The authors declare that all other relevant data supporting the findings of this study are available within the article and its Supplementary Information files, or from the corresponding authors upon request.

Field-specific reporting

Please select the one below that is the best fit for your research. If you are not sure, read the appropriate sections before making your selection.

☒ Life sciences ☐ Behavioural & social sciences ☐ Ecological, evolutionary & environmental sciences

For a reference copy of the document with all sections, see nature.com/documents/nr-reporting-summary-flat.pdf

Life sciences study design

All studies must disclose on these points even when the disclosure is negative.

Sample size	All the experiments were performed in triplicate because dispersion of the values in our experiments were not large.
Data exclusions	No data were excluded from the analyses.
Replication	All attempts at replication were successful.
Randomization	This is not relevant to our study because this is the association of a fungal-endobaterial symbiont.
Blinding	Investigators were not blinded to analyses in our study because those in this study (e.g. measurements of antibacterial activities) are not affected by cognitive biases.

Reporting for specific materials, systems and methods

We require information from authors about some types of materials, experimental systems and methods used in many studies. Here, indicate whether each material, system or method listed is relevant to your study. If you are not sure if a list item applies to your research, read the appropriate section before selecting a response.

Materials & experimental systems

n/a	Involved in the study	
☒	☐	Antibodies
☒	☐	Eukaryotic cell lines
☒	☐	Palaeontology and archaeology
☒	☐	Animals and other organisms
☒	☐	Human research participants
☒	☐	Clinical data
☒	☐	Dual use research of concern

Methods

n/a	Involved in the study	
☒	☐	ChIP-seq
☒	☐	Flow cytometry
☒	☐	MRI-based neuroimaging
